# Site effects how-to and when: An overview of retrospective techniques to accommodate site effects in multi-site neuroimaging analyses

**DOI:** 10.3389/fneur.2022.923988

**Published:** 2022-10-31

**Authors:** Johanna M. M. Bayer, Paul M. Thompson, Christopher R. K. Ching, Mengting Liu, Andrew Chen, Alana C. Panzenhagen, Neda Jahanshad, Andre Marquand, Lianne Schmaal, Philipp G. Sämann

**Affiliations:** ^1^Centre for Youth Mental Health, University of Melbourne, Melbourne, VIC, Australia; ^2^Orygen, Parkville, VIC, Australia; ^3^Imaging Genetics Center, Mark and Mary Stevens Neuroimaging and Informatics Institute, Keck School of Medicine, University of Southern California, Marina del Rey, CA, United States; ^4^School of Biomedical Engineering, Sun Yat-sen University, Shenzhen, China; ^5^Department of Biostatistics, Epidemiology, and Informatics, Penn Statistics in Imaging and Visualization Center, University of Pennsylvania, Philadelphia, PA, United States; ^6^Center for Biomedical Image Computing and Analytics, University of Pennsylvania, Philadelphia, PA, United States; ^7^Programa de Pós-graduação em Ciências Biológicas: Bioquímica, Universidade Federal do Rio Grande do Sul, Porto Alegre, Brazil; ^8^Department of Translational Psychiatry, Max Planck Institute of Psychiatry, Munich, Germany; ^9^Laboratory of Brain eScience, Mark and Mary Stevens Neuroimaging and Informatics Institute, Keck School of Medicine of USC, University of Southern California, Marina del Rey, CA, United States; ^10^Department of Cognitive Neuroscience, Donders Institute for Brain, Cognition and Behavior, Radboudumc, Nijmegen, Netherlands; ^11^Max Planck Institute of Psychiatry, Munich, Germany

**Keywords:** MRI, multi-site study, ComBat, normative modeling, site effect, neuroimaging, deep learning, generative adversarial networks (GANs)

## Abstract

Site differences, or systematic differences in feature distributions across multiple data-acquisition sites, are a known source of heterogeneity that may adversely affect large-scale meta- and mega-analyses of independently collected neuroimaging data. They influence nearly all multi-site imaging modalities and biomarkers, and methods to compensate for them can improve reliability and generalizability in the analysis of genetics, omics, and clinical data. The origins of statistical site effects are complex and involve both *technical* differences (scanner vendor, head coil, acquisition parameters, imaging processing) and differences in *sample characteristics* (inclusion/exclusion criteria, sample size, ancestry) between sites. In an age of expanding international consortium research, there is a growing need to disentangle technical site effects from sample characteristics of interest. Numerous statistical and machine learning methods have been developed to control for, model, or attenuate site effects – yet to date, no comprehensive review has discussed the benefits and drawbacks of each for different use cases. Here, we provide an overview of the different existing statistical and machine learning methods developed to remove unwanted site effects from independently collected neuroimaging samples. We focus on linear mixed effect models, the ComBat technique and its variants, adjustments based on image quality metrics, normative modeling, and deep learning approaches such as generative adversarial networks. For each method, we outline the statistical foundation and summarize strengths and weaknesses, including their assumptions and conditions of use. We provide information on software availability and comment on the ease of use and the applicability of these methods to different types of data. We discuss validation and comparative reports, mention caveats and provide guidance on when to use each method, depending on context and specific research questions.

## Introduction

Multi-site data analysis is now the typical practice in consortium projects such as the Enhancing NeuroImaging Genetics through Meta-Analysis (ENIGMA) consortium (1), the Cohorts for Heart and Aging Research in Genomic Epidemiology (CHARGE) consortium (2), iSTAGING (3) as well as in other large scale data aggregation efforts (4–6). These efforts aim to pool together data and statistical information across independent studies collected across a wide range of locations and study designs. However, the distribution of extracted features can be heavily dependent on the site at which it was collected, and these site effects represent a considerable statistical challenge for neuroimaging analyses. Retrospective site-correction techniques are now common-place for multisite neuroimaging analyses, yet knowing which method to use under what conditions is not always clear. The focus of our review, therefore, is to provide an overview of the methods to date and provide the first ever set of site-correction guidelines.

Several goals motivate the use of multi-site data both for hypothesis-driven and exploratory analyses: one goal is to increase *statistical power* by increasing the total number of observations. This improves the ability to detect small but likely true effects. Another goal is to boost the *generalizability* and hence the scientific value of the results by directing the same hypothesis to independent cohorts. Similarly, multivariate pattern analysis (MVPA) needs large, heterogeneous and representative data to effectively detect subgroups (“biotyping”) and to develop classification tools through supervised machine learning that generalize well to unseen data (7, 8). In this line, MVPA with small sample sizes tends to lead to inflated accuracy estimates during cross validation (9). To attenuate dataset shift issues in machine learning, it has actually been recommended to allow for more clinical heterogeneity (8), for example, include patients with different ethnicity or from different age classes. As a natural consequence, this inclusion strategy enhances site effects that need to be considered to make optimal use of large multisite samples (10). Both power and generalizability also play a role in analyses that need certain variables stretched out over a sufficiently large range. These include, for example, lifespan studies of brain development and aging that benefit from concatenating studies to cover a large age range (11–13). Finally, *open data sets* often contain multi-site data, representing a pool of neuroimaging data sets have been collected as part of different studies and then gathered.

In the light of these goals, one statistical challenge is to estimate the variance explained by the (acquisition) site without impacting the model's capacity to estimate other effects that co-vary with the site. Insufficient correction for site effects can leave serious confounds in the data that obscure interpretations, impair the generalizability, replication and reproducibility and hinder multivariate approaches such as MVPA and machine learning (ML). In turn, overcorrection of site effects may interfere with the estimation of effects of interest and the effects of confounders or other nuisance variables that are needed for a valid model (e.g., age effects) (14).

What sources of variability exist that contribute to “site-effects” and affect the imaging phenotypes at a specific site systematically? For magnetic resonance imaging (MRI), which is the primary topic of this article, the MR imaging platform, its field strength, properties of the main magnet, gradient coils and receiving coils and the sequence specification, such as voxel size, are all examples of major sources of different raw signal properties that propagate into the derived features (15, 16). Site differences are even more heterogeneous for functional MRI due to additional factors such as variable acquisition geometry and brain coverage, head coils, bias fields (17), difference in task implementation, temporal duration, EPI based sequences and complex effects of motion (18) with static and motion-dependent image distortions and signal dropouts (19). A site's (re-)positioning strategy (20, 21) or the use of immobilization devices may even influence the uniformity of the acquisitions from one participant to the next. The complexity increases in multisite task-based functional MRI due to its interactive element: Here, details of the task instructions, circadian effects and other procedural details may influence site-specific differences in the behavior of the participants, even for low-demand tasks such as resting state fMRI (rs-fMRI) (22). In multi-site analyses such as those practiced by the ENIGMA consortium, considerable effort is made to standardize image processing protocols (http://enigma.ini.usc.edu/protocols/imaging-protocols). In addition, data quality control (QC) procedures that often cannot be fully automated but require a qualitative assessment by a reader, can be standardized (23). Further, to reduce influences of different (combinations of) software versions, containerized, user-friendly software packages are often distributed (24).

**Figure 1:**
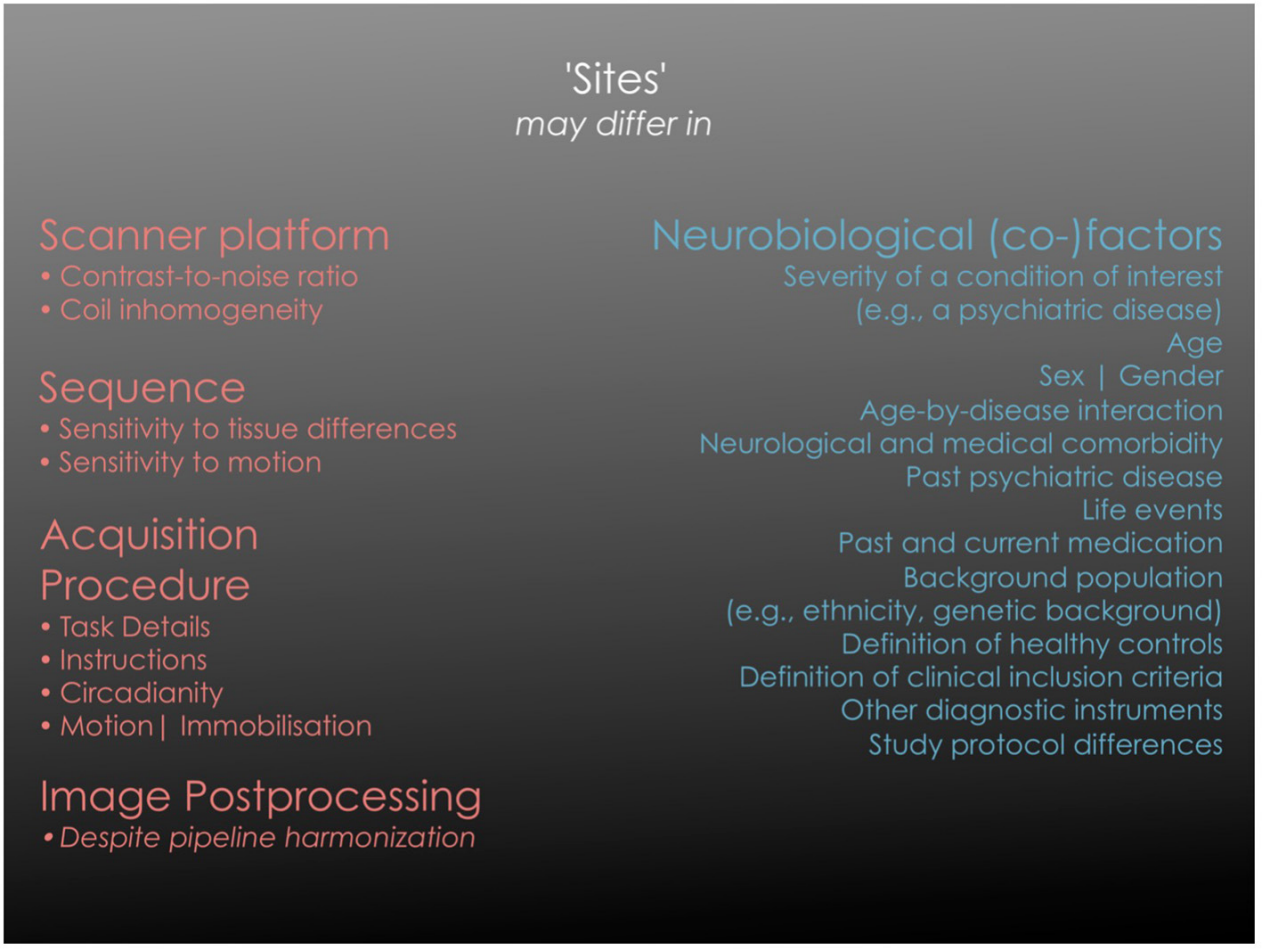
Overview of different sources of site effects. The **left** column lists categories of technical factors that are bound to a specific acquisition site and that may significantly influence the primary image properties. “Scanner effect” is often used as an abbreviation for “image acquisition platform” in a wider sense while it incorporates numerous technical components. In active tasks where researchers or technicians intervene as part of the study protocol (e.g., provide instructions to participants), additional site specific effects of a systemic nature may appear. The **right** column lists factors that may be variables of interest (such as a disease status) or variables of no interest, depending on the study question. Each of them may be site-specific and thus co-vary with the specific site in a multi-site analysis, imposing the challenge of disentangling the two sources of “site effects”. It should be noted that “variables of no interest” may still be essential for an adequate statistical model of the biological effects (e.g., age).

Sample characteristics are the other major source of site effects, as “site” and “sample/cohort” are often indistinguishable. Sample characteristics comprise the population from which participants are sampled, or the criteria by which healthy controls are defined, including tolerance thresholds for current or past illness. In clinical samples, diagnostic criteria, their operationalization and potentially ill-defined context variables, as well as inclusion and exclusion criteria have an influence. Cohort characteristics are thus the most disputable source of site related variability that must be handled carefully because they may carry true variability of the effect of interest. We give an overview over potential sources of site difference in Figure 1.

The imaging trait, or phenotype, can originate from a broad range of modalities and carry different levels of abstraction, yet as long as it is linked to a common anatomical or functional reference or index system, it is analyzable *across subjects*. Fully automated analyses such as by the FreeSurfer segmentation pipeline (http://surfer.nmr.mgh.harvard.edu) lead to regional cortical thickness, surface area, cortical volume, or gyrification indices, and many of these features are also available at the vertex level. Voxel-based analyses generate measures in stereotactic atlas space with increasingly precise intersubject coregistration (25–27). Task-based fMRI is meanwhile routinely contained for large cohort studies (28) or deep phenotyping studies (29), and the same is true for features from structural or functional connectomics with bi-regional connectivity, network specific connectivity metrics (30) or global or regional graph theory based network properties (31). The statistical principles of the site effect correction methods are generally valid and applicable to all types of human brain mapping data as long as the features refer to a common anatomical or functional reference system which can be indexed across different subjects.

### Overview and structure of this manuscript

Several strategies exist to handle site effects, including statistical methods performed on derived features, and more deep learning based tools that tend to adjust site effects image-wide. We explain and discuss the statistical tools that are applied *post-hoc* to imaging metrics in two groups: A first group that uses regression models to remove variance such as the adjusted residuals harmonization (32–34) and the ComBat approach that modifies the regression approach to preserve defined covariate system (11, 33, 34). We also present variants of ComBat such as CovBat that harmonizes site-specific covariance patterns across features (35), implementations for longitudinal data (36) or non-linear covariate effects (35), or approaches that estimate the ComBat correction parameters from low level image characteristics, such as. image quality metrics (37). The second category is normative modeling that maps rather than adjusts the metrics given their site and covariate information (38–40). Last, we look into deep learning algorithms that transform the raw images based on learned, virtual, site-specific prototypes (41–44). In Section Overview of site effect correction tools we describe the statistical foundation of currently available methods, their degree of validation, software availability, computational demands, and key advantages and disadvantages, such as the possibility to transfer the model to unseen cases or sites, or the possibility to choose a reference site. We acknowledge that the list of methods discussed in this paper is not exhaustive but chosen based on their level of availability, dissemination and theoretical considerations. In Section Discussion we discuss comparative reports, highlight caveats for the use of some methods and inform on considerations that might guide the selection of an appropriate method to attenuate site effects.

### Who should read this manuscript

This review will be most helpful in academic settings in which researchers are analyzing multi-site neuroimaging data—for example, from a publicly available repository or in a consortium. In fact, we hope that this manuscript will encourage the use of open, shared pooled data sets by providing an overview and guidance over several tools to accommodate site effects and by facilitating their use. Yet, site effects are generic and can be found in many settings—spanning from behavioral data analyzed in interventional clinical trials to cohort studies in social sciences, to gene expression or any other biological data with site or batch effects—and so, the statistical principles presented here are not specific to imaging phenotypes as dependent variables. To address readers from different scientific backgrounds with different degrees of statistical knowledge and interest, we present the methods at different levels of granularity: for a short summary of each method, the reader may want to focus on the sections regarding the advantages and disadvantages of each approach and the general discussion. For the more methodologically-curious readers, the statistical sections on each method provide a deeper insight into the derivation of each method.

## Overview of site effect correction tools

In the following sections we focus on methods that consider the presence of covariate effects in their theoretical conceptualization. We do not describe in detail simple global scaling (32, 33), residuals harmonization (33) or the surrogate variable analysis (SVA) approach that after dimensionality reduction incorporates component weights as nuisance variables (45).

**Figure 2:**
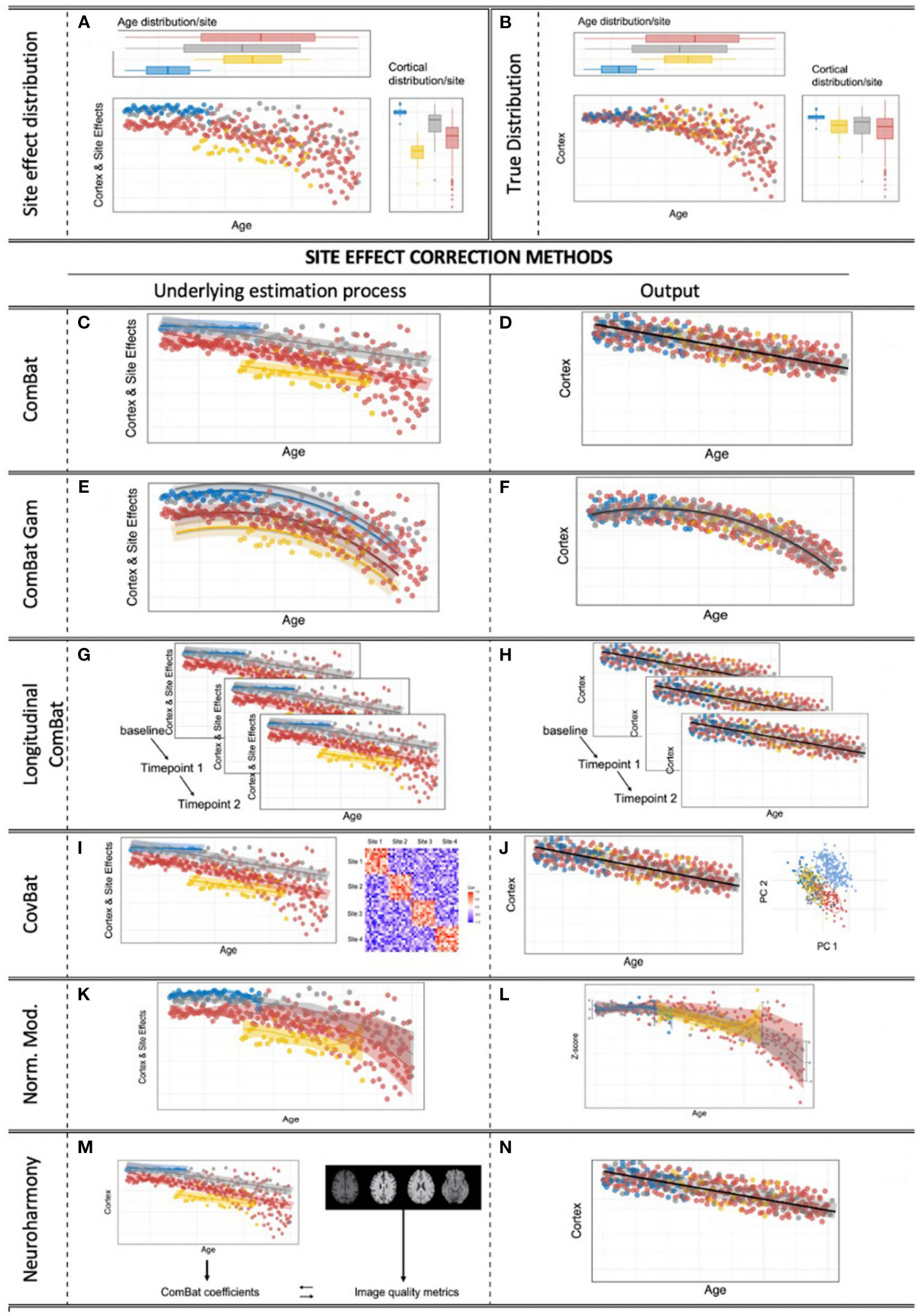
Site effects & correction methods for multi-site effects in neuroimaging. **(A)** Obvious site effects with preserved age effect within the sites. **(B)** True, underlying, unknown heteroscedastic distribution. **(C,D)** The standard ComBat algorithm provides linear adjustment of site means and scaling of site variance differences, with the option to preserve linear covariate effects of interest, while scaling the variance to a homoscedastic distribution. **(E,F)** ComBat-GAM: ComBat is augmented by the option to expand defined covariates of interest by penalized non-linear expansion terms, while scaling the variance to a homoscedastic distribution. **(G,H)** Longitudinal ComBat: The ComBat algorithm is modified to model within-subject variance over several time points under the additional consideration of changing (linear) covariates. **(I,J)** CovBat: After application of the original ComBat algorithm, ComBat is again applied to the principal components of the residuals to harmonize site-specific covariance. **(K,L)** Normative modeling allows the user to convert raw engineered features into z-scores specifically adjusted to separately model site effects and covariate effects, all under a Bayesian prior system. **(M,N)** *Neuroharmony* allows one to harmonize features of a single subject based on raw T1-image based quality metrics that have previously been linked to ComBat correction coefficients by a supervised ML algorithm that has been trained on a large neuroimaging data set.

An illustrative overview over all methods is given in Figure 2. In addition, we have provided a summary table including details on the number of participants, sites, the type of feature that each method was validated on for all papers cited in this manuscript (Supplementary Table S1).

### Notation

For the convenience of the reader, we aim to provide a consistent selection of parameter names and the notation of equations and mathematical symbols. We follow general notation practices, such as denoting the mean of x by as $\bar{x}$, an estimate of x by $\hat{x}$ and a new value or prediction of x by *x*_∧_*, if not noted otherwise. Running indices follow standard practices, such as that *x*_*isf*_ represents the value of subject *i*, site *s* and feature *f*. Lowercase letters, such as *x*, represent single values, bold lowercase letters **x** denote vectors, and uppercase letters *X* matrices. Vectors are column vectors if not noted otherwise. Furthermore, we adopted the notation and parameter naming of the original authors (which is often also reflected in the code implementing a method) as much as possible, but have made adjustments for consistency within this document and the flow of the text. Lastly, the difference between standardized and unstandardized values is often marked by selecting different parameters (in papers of the ComBat family standardized values are represented by *Z*, unstandardized values by *Y*). For readability and the convenience of the reader, we abstain from this differentiation and note at this point that all values in the following need to be considered as standardized.

### Adjusted residuals harmonization

One very simple harmonization technique is adjusting the observed values by a simple estimate of the residuals from a multiple regression analysis that includes site and a set of covariates as predictors. The even simpler approach—of just regressing out site effects without considering a covariate system—is not recommended (33, 38).

#### Statistical foundation

This approach is based on the additive modeling of site effects and covariate effects:


$$\begin{array}{l} Y_{isf}=\alpha_{f}+X\beta_{f}+\gamma_{sf}+\epsilon_{isf} \end{array}$$

with *f* being the feature index, *s* being the site index, α_*f*_ the grand mean of the feature across sites, *X* represents the covariate matrix, **β**_*f*_ the coefficients associated with *X* and **γ**_*sf*_ the site effect parameters. The residual terms ϵ_*isf*_ are assumed to have a mean of 0. Using ordinary least squares (OLS), for each feature *f*, the estimators ${\hat{\beta }}_{f}$ and ${\hat{\gamma }}_{sf}$ are obtained. The adjustment is performed by subtracting the site related term:


$$\begin{array}{l} Y_{isf}^{Adj}=Y_{isf}-{\hat{\gamma }}_{sf}. \end{array}$$

#### Advantages and disadvantages

Adjusted residuals harmonization can be implemented easily in every statistical environment that allows for multiple regression analysis. As described above, covariate effects β_*f*_ are considered in the way that during the OLS procedure the covariate effects “compete” with the site effects and attenuate their correlation with the dependent data. However, the approach has several disadvantages: first, multicollinearity between site and covariates may lead to unstable estimations of the regression coefficients and to both undercorrection or overcorrection of site and covariate effects. Second, different scaling behaviors of the sites (i.e., different variance of the same features between scanners) are not modeled. Particularly when aiming to combine independently collected data, we advise against using this procedure, as the variance retained in *X*_*is*_β_*f*_ is strongly dependent on the covariate effects and the degree of collinearity between covariates and sites. Nygaard et al. (46) demonstrated that in unbalanced samples, the residualization approach (two-factorial ANOVA in their example) leads to overconfident group results.

### The ComBat approach

The ComBat approach was originally suggested for microarray gene expression data (34); its main purpose was to improve the location/scale model (47) for small samples. Applied to neuroimaging data, the ComBat approach assumes a multisite dataset with “site” often representing “scanner” in the sense of systematically different imaging acquisition platforms. It then builds on two principles: first, a site-specific shift and a site-specific scaling factor is assumed and estimated by a Bayesian approach. In detail, this Bayesian approach generalizes the OLS approach outlined above, adding empirical priors over the site specific means and variance, which results in partial pooling over the features (genes in the original implementation, but voxels or regions in neuroimaging). Second, covariates (that may be confounded with site) can be defined in order to incorporate their effect on the variance and preserve between-subject biological variability. ComBat was first developed and validated for diffusion tensor imaging data—more specifically, for maps of fractional anisotropy values (32); later, cortical thickness measurements were analyzed (33). The tool is widely used, and since 2017, over 50 imaging studies refer to ComBat as their site effect correction approach (48). Below, we describe standard ComBat and also conceptual extensions: ComBat-GAM provides an improved model for nonlinear covariate effects (11), longitudinal ComBat refines the modeling of serial measurements (36), and distributed ComBat (49) allows to harmonize data locally without exchanging raw features. Finally, CovBat builds on ComBat and in addition harmonizes the covariance pattern of the residuals (49).

#### Statistical foundation

As its starting point, the ComBat algorithm assumes that an individual's observed score *Y*_*isf*_ for site *s*, individual *i* and feature *f* is a combination of a site effect and components not associated with the site:


$$\begin{array}{l} Y_{isf}=\alpha_{f}+X\beta_{f}+\gamma_{sf}+\delta_{sf}\;\epsilon_{isf} \end{array}$$

The non-site effect related components include: α_*f*_, the overall mean per feature; β_*f*_ the corresponding coefficients to the covariate matrix *X*; and the error term ϵ_*isf*_ with an expected mean of 0 and site specific variance multiplier $\sigma_{f}^{2}$. The site-effect related components include γ_*fs*_, an (additive) offset from the grand mean per site per feature, and δ_*sf*_, a (multiplicative) effect affecting the dispersion around the mean, thus changing the variance of the error term to δ_*sf*_ ϵ_*isf*_.

The ComBat algorithm is based on a location and scale (L/S) adjustment, a procedure in which these additive effects (location, mean) and multiplicative effects (scale, variance) are removed per feature per site by subtracting the additive effects and dividing by the multiplicative site effects. To do this, ComBat can be estimated in two ways: *mean only* in which the estimates for mean ${\hat{\gamma }}_{sf}$ and variance ${\hat{\delta }}_{sf}$ for site *s* and feature*f*are estimated from the sample (mean as best estimator of the mean, pooled variance as best estimator of the variance), and in an *empirical Bayes (EB)* mode, in which those best estimators (labeled $\gamma_{sf}^{*}$ and $\delta_{sf}^{*}$) are obtained by a fusion between ${\hat{\gamma }}_{sf}$ and ${\hat{\delta }}_{sf}$ and a prior distribution:


$$\begin{array}{l} \gamma_{sf}\sim \left(\gamma_{s},\tau_{s}^{2}\right)\text{ and }\delta_{sf}^{2}\sim \Gamma \left(\lambda_{s},\;\theta_{s}\right) \end{array}$$

The hyperparameters ${\hat{\gamma }}_{s},{\hat{\tau }}_{s}^{2},{\hat{\lambda }}_{s},{\hat{\theta }}_{s}$ are estimated by assuming the site *s* sample mean for feature *f* is the mean of all individuals in this sample for this feature: ${\hat{\gamma }}_{sf}$=$\frac{1}{n_{s}}\sum_{i}Y_{sfi}$. Concluding, the estimates of ${\bar{\gamma }}_{s}$ (the overall mean per site across the number of all features, *F*) and the corresponding variance $\tau_{s}^{2}$ of those site *s* sample means γ_*sf*_ from ${\bar{\gamma }}_{s}$ are given by (respectively):


$$\begin{array}{l} {\bar{\gamma }}_{s}=\frac{1}{F}\sum_{f}{\hat{\gamma }}_{sf},\text{ and }\tau_{s}^{2}=\frac{1}{F-1}\sum_{f}{\left({\hat{\gamma }}_{sf}-\;{\bar{\gamma }}_{s}\right)}^{2}. \end{array}$$

In addition, assuming that ${\hat{\delta }}_{sf}^{2}=\frac{1}{n_{s}-1}\sum_{i}{\left(Y_{isf}-{\hat{\gamma }}_{sf}\right)}^{2}$ (sample variance for site *s* and feature *f*) we can deduce that the mean ${\bar{V}}_{s}$ per site *s* of those ${\hat{\delta }}_{sf}^{2}$ across the number of all features, *F*, is ${\bar{V}}_{s}=\frac{1}{F}\sum_{f}{\hat{\delta }}_{sf}^{2}$ and the corresponding variance ${\bar{S}}_{s}^{2}=\frac{1}{F-1}\sum_{f}{\left({\hat{\delta }}_{sf}^{2}-{\bar{V}}_{s}\right)}^{2}$. The sample moments of ${\bar{V}}_{s}$ and ${\bar{S}}_{s}^{2}$ are then set against the theoretical moments of the *Inverse* Γ distribution with the mean $\frac{\theta_{s}}{\lambda_{s}-1}$ and variance $\frac{\theta_{s}^{2}}{{\left(\lambda_{s}-1\right)}^{2}\left(\lambda_{s}-2\right)}$. Solving for $\bar{\lambda_{s}}$ and ${\bar{\theta }}_{s}$ results in estimates those hyperparameters as follows:


$$\begin{array}{l} \bar{\lambda_{s}}=\frac{{\bar{V}}_{s}+2{\bar{S}}_{s}^{2}}{{\bar{S}}_{s}^{2}}\text{ and }{\bar{\theta }}_{s}=\frac{{\bar{V}}_{s}^{3}+{\bar{V}}_{s}{\bar{S}}_{s}^{2}}{{\bar{S}}_{s}^{2}}. \end{array}$$

Estimating the parametric batch effect adjustments

We place prior distributions over γ_*sf*_ and $\delta_{sf}^{2}$ which we assume to have the shape:


$$\begin{array}{l} \gamma_{sf}\;\sim \;N\left({\hat{\gamma }}_{s},\tau_{s}^{2}\right)\text{ and }\delta_{sf}^{2}\;\sim \;\text{Inverse }\Gamma \left(\lambda_{s},\theta_{s}\right). \end{array}$$

For both parameters we aim to find the posterior conditional distributions for γ_*sf*_ and $\delta_{sf}^{2}$, which are denoted $\pi \left(\gamma_{sf}|Y_{sf},\delta_{sf}^{2}\right)$ and $\pi \left(\delta_{sf}^{2}|Y_{sf}\gamma_{sf}\right)$, respectively.

Applying Bayes' theorem, it can be shown that the expected values for $\gamma_{sf}^{*}$ and $\delta_{sf}^{2^{*}}$ are equal to (see detailed derivation in the Appendix):


$$\begin{array}{l} \gamma_{sf}^{*}=Ê\left[\gamma_{sf}|Y_{sf},\sigma_{sf}^{2^{*}}\right]=\frac{n_{s}{\bar{\tau }}_{s}^{2}{\hat{\gamma }}_{sf}+\delta_{sf}^{2}{\bar{\gamma }}_{s}}{n_{s}{\bar{\tau }}_{s}^{2}+\;\delta_{sf}^{2^{*}}}\\ \delta_{sf}^{2*}\;=\;Ê\left[\delta_{sf}^{2}|Y_{sf},\gamma_{sf}^{*}\right]\;=\;\frac{{\bar{\theta }}_{s}\;+\;\frac{1}{2}\sum_{i}{\left(Y_{isf}-\gamma_{sf}^{*}\right)}^{2}}{\frac{n_{i}}{2}\;+\;{\bar{\lambda }}_{s}-1} \end{array}$$

As we can see from the estimates for $\gamma_{sf}^{*}$ and $\delta_{sf}^{2^{*}}$, the two parameters are not independent from each other - the system does not have an exact analytical solution. Hence, the estimates for the parameter values of $\gamma_{sf}^{*}$ and $\delta_{sf}^{2^{*}}$ need to be found iteratively, by starting with reasonable values for $\gamma_{sf}^{*}$ and $\delta_{sf}^{2^{*}}$ and looping over several sets of parameter values and under the constraint of a loss function - for example, within an expectation maximization (EM) algorithm (50).

The rationale behind the EB step and creating this prior is its robustness toward outliers, particularly in smaller samples, by borrowing information from all features for the variance estimate. As the EB adjustment diverges from the prior with increasing sample size, this results in comparable results (to the L/S adjustment) for medium sample sizes, but results in strong adjustments for small sample sizes, protecting the site effect mean and variance estimates from the influence of outliers (34).

Based on the estimates of of ${\hat{\gamma }}_{sf}$ and ${\hat{\delta }}_{sf}^{2}$, ComBat returns adjusted data $Y_{isf}^{*}$:


$$\begin{array}{l} Y_{isf}^{*}=\frac{Y_{isf}-{\hat{\alpha }}_{f}-X{\hat{\beta }}_{f}-{\hat{\gamma }}_{sf}}{{\hat{\delta }}_{sf}}+{\hat{\alpha }}_{f}+X{\hat{\beta }}_{f}, \end{array}$$

where ${\hat{\alpha }}_{f},{\hat{\gamma }}_{sf},{\hat{\beta }}_{f}$ and ${\hat{\delta }}_{sf}$ are the respective parameter estimates. These parameter estimates can be used to apply the correction model to the data per feature *f* and site *s*. Beyond this, yet depending on the implementation, ComBat allows the user to either align data from all sites to the (grand) mean and (pooled) variance of all sites or to that of a reference site (see Table 1).

**Table 1:** List of tools and respective open source code links.

**Method**	**Code**	**Link to software**	**Reference**	**Comment**
ComBat	R, Matlab	https://github.com/Jfortin1/ComBatHarmonization	(32)	Reference batch function: possible in Python and R
	Python	https://github.com/Jfortin1/ComBatHarmonization		
ComBat	R	http://enigma.ini.usc.edu/protocols/statistical-protocols	(51)	Added functions: separate into train|apply, and handling of missing values
ComBat	Python	https://github.com/brentp/combat.py	(34)	
ComBat	Python	https://github.com/Warvito/neurocombat_sklearn	(32)	
M-ComBat	R	https://github.com/SteinCK/M-ComBat	(52)	Modified ComBat allowing for harmonization on a reference sample
d-ComBat	R	https://github.com/andy1764/Distributed-ComBat	(49)	Distributed ComBat allowing to apply ComBat w/o exchange of original site data
ComBat-GAM	Python	https://github.com/rpomponio/neuroHarmonize	(11)	Additional scripts for working with NIFTI
Longitudinal ComBat	R	https://github.com/jcbeer/longCombat	(36)	
CovBat	R, Python	https://github.com/andy1764/CovBat_Harmonization	(35)	Includes a ComBat version allowing to separate into train|apply
Neuro-harmony	Python	https://github.com/garciadias/Neuroharmony; https://mriqc.readthedocs.io	(37)	Second tool (MRIQC) needed to generate quality measures
Hierarchical Bayesian Regression	Stan	https://github.com/likeajumprope/Bayesian_normative_models	(38)	NM focusing on site effect correction
Normative modeling toolbox	Python	https://github.com/amarquand/PCNtoolkit	(53)	Toolkit on NM functions

The following table lists the tools discussed in this paper, the key paper that informs on the statistical background and the link to the open source codes.

Deep learning tools are not listed here due to their more complex handling and still ongoing validation studies.

#### Advantages and disadvantages

ComBat was validated in the two reports that introduced it to neuroimaging (32, 33) and since then has been used in more than 50 peer-reviewed neuroimaging studies. Further validation comes from studies that compared it to other methods (see discussion for details). The computational effort of ComBat is low. Also, for higher dimensional data types such as voxel-based morphometry, implementations exist in R, Matlab and Python, reflecting its broad acceptance by neuroimaging researchers. In the following sections we discuss some implications that follow from the ComBat framework and considerations that should be kept in mind when applying ComBat:

As outlined above, the Bayesian step poses empirical priors over the estimates for mean and variance, leading to a partial pooling of information over the features in the data set. This has two direct implications: First, it implicitly assumes that signal properties of the different features are drawn from the same distribution with a single mean and variance for each site. However, this assumption does not hold if the target distribution is heteroscedastic, meaning that the standard deviation of the predicted variable is not consistent (for example cortical thickness showing greater variance in old than in young individuals). Second, sites with less data are more heavily regularized than those with more data, leading to unbalanced adjustments in case of large sample size differences between sites.

Both implications are a direct result from the modeling of modeling noise in ComBat: within the ComBat framework, the variance term ϵ_*isf*_ is modeled as an empirical average of the variance from all data. This forces all sites to have the same (homoscedastic) noise term ϵ_*isf*_ and leads to a loss of the biological meaning that is included in heteroscedasticity (e.g as described in the example above). Instead, the modeling of the noise term in ComBat leads to ϵ_*isf*_ becoming sample size dependent: The ϵ_*isf*_ in a data set with two sites with *n*_1_ = 10000 samples and *n*_2_ = 100 will be different from the ϵ_*isf*_ of two sites with *n*_1_ = 500 and *n*_2_ = 500. The limitation to a unified, average noise term within the ComBat framework also prevents the approach to cater for the differences in precision that result from site sample differences as described in the second implication. To conclude, Combat is not able to model heteroscedastic distributions and large sample differences between sites, and should not be used in those cases.

Another disadvantage is that while covariate effects can be preserved by ComBat, they need to be made explicit to the algorithm. Covariate effects might be collinear with site but that are not specified (due to unavailability of the covariate or due to not considering its influence) are not preserved and variability of the imaging phenotypes explained by this (unmodelled) covariate will be removed from the total variance. Further, ComBat assumes that the site effects ${\hat{\gamma }}_{sf}$ and ${\hat{\delta }}_{sf}^{2}$ are independent of the covariate effects modeled in $X{\hat{\beta }}_{f}$, and does not model site-by-covariate interactions. This suggests that ComBat is optimally applicable when the biological effects that are assumed to have an effect on ${\hat{\gamma }}_{sf}$ and ${\hat{\delta }}_{sf}^{2}$ are equally distributed across sites and can thus be estimated across all subjects. In scenarios with a strong collinearity between covariates *X* and a site (e.g., one site containing a group of young subjects, yet with very low cortical thickness values due to the scanner of that site), problems occur: First, the algorithm may force the sample to align with a generally assumed linear age trajectory, i.e., strongly increasing the values of this site. Second, this shift may be particularly strong if no non-linear age effects are modeled over the life-span (e.g., allowing for a stable plateau phase in young adulthood). In this respect, Pomponio et al. (11) recommended – based on simulation experiments – that covariate ranges should ideally not be disjoint but should overlap between sites.

As a practical disadvantage, different implementations of ComBat with different functionality exist in parallel: for example, the version of Fortin et al. (32) cannot process input data with missing values or fit the model in a training dataset and apply it to a test dataset. Both functions were added to the version published by Radua et al. (51). Similarly, not all implementations allow the user to define a subsample as a training sample in a machine learning context (see Table 1).

### Combat-GAM

#### Statistical foundation

ComBat-GAM (Pomponio et al., 2020) allows for modeling of non-linear covariate effects by placing one covariate or a set of covariates within a function *f*(*x*):


$$\begin{array}{l} Y_{isf}^{*}=\frac{Y_{isf}-f\left(X_{isf}\right)-{\hat{\gamma }}_{sf}}{{\hat{\delta }}_{sf}}\;+\;f\left(X\right) \end{array}$$

where **f**(*X*_*isf*_) is a smooth function over covariates *x*_1_, *x*_2_, *x*_3_, …, *x*_*n*_ with **f**(*X*_*isf*_) = **f**(*x*_1_, *x*_2_, *x*_3_, …, *x*_*n*_) = *a* + *f*_1_(*x*_1_) + *f*_2_(*x*_2_)+*f*_3_(*x*_3_)+, …, +*f*_*n*_(*x*_*n*_). Very briefly [for an extensive introduction and overview, see Wood (54)], the GAM approach used by Pomponio et al., (11) divides the input into sections and defines a spline, i.e., a piecewise polynomial fit to the data on these sections. The points separating the splines are called *knots*. *f*(*x*) is then represented by a basis expansion by choosing a *basis* (a space of functions) that *f* (or a close approximation to it) is an element of. One *basis function* is then piecewise fit to each spline, overall approximating *f*. In the simplest case, the basis function is linear, but more complex structures have been introduced. Furthermore, smoothness terms are added that penalize the “wiggliness” of the fitted basis functions, to prevent overfitting. Beyond that, the use of GAMs has led to complex ways of optimizing the placement of knots and interpolating over several predictors.

To model non-linear age effects, as a prerequisite for lifespan studies, Pomponio et al., (11) used plate regression splines for the basis expansion, a method that optimizes the knot placement problem over several covariates. Model estimation was performed using penalized regression splines with the smoothness defined from the restricted maximum likelihood (REML) criterion. Pomponio et al., (11) included age as non-linear term and sex and ICV as linear terms in the model, reducing it to *f*(*x*_*age*_, *x*_*sex*_, *x*_*ICV*_) = α + *f*_1_(*x*_*age*_) + *b*_2_*x*_*sex*_ + *b*_3_*x*_*ICV*_.

#### Advantages and disadvantages

The main advantage of ComBat-GAM is the option to model and thereby preserve non-linear covariate effects in a flexible way during the site correction process. Pomponio et al., (11) present its performance on several brain volume phenotypes over a large age range, and differently shaped non-linear age trajectories were detected in many regions (see discussion for comparative studies). The tool is available as a python script package and is easy to use. It also allows a user to estimate the model in a subsample (“training sample”) and apply it to other examples from the same sites. Further, besides feature tables (e.g., cortical thickness of regions of interest), 3D images in NIFTI format can be used as input data, making ComBat-GAM useful for higher-dimensional data, for which its computational performance is also sufficient.

In terms of disadvantages, the method inherits the general limitations of ComBat. Mainly, ComBat-GAM cannot preserve effects of site-confounded covariates that are not specified. Another problem might be overfitting of non-linear effects which could hamper the transfer of the model to unseen cases.

### CovBat

CovBat (49) extends the ComBat approach toward harmonizing scanner-specific mean and variance of all features separately, but also the covariance structure between features. This additional function aims particularly to accommodate for site effects in preparation for Multivariate Pattern Analysis (MVPA), a method that analyzes the joint distribution and correlation structure among multiple brain features instead of univariate features.

### Statistical foundation

CovBat first calculates the ComBat-adjusted residuals, by running the default version of ComBat:


$$\begin{array}{l} e_{isf}=\frac{Y_{isf}-{\hat{\alpha }}_{f}-X{\hat{\beta }}_{f}-\;{\hat{\gamma }}_{sf}}{{\hat{\delta }}_{sf}} \end{array}$$

After this first step, the *e*_*isf*_ are assumed to have a mean of 0, however, their covariance matrices Σ_*i*_ may still differ across sites. In a second step, principal components analysis (PCA) is performed on those residuals *e*_*isf*_. PCA identifies covariance patterns in high dimensional data and compresses them by reducing the number of its dimensions. Conceptually, PCA consists of finding *q* orthogonal axes ϕ_*k*_:

${\hat{\lambda }}_{ik}=\overset{n_{i}}{\underset{j=1}{\sum }}{\left(\xi_{ijk}-\overset{n_{i}}{\underset{j=1}{\sum }}\xi_{ijk}/n_{i}\right)}^{2}/\left(n_{i}-1\right)$. To summarize, site differences create variation of principal component scores from the average. Based on these assumptions, the site effects can be removed via $\xi_{isk}^{CovBat}=\frac{\xi_{isf}-{\hat{\mu }}_{sk}}{{\hat{\rho }}_{sk}}$. As a last step, the CovBat adjusted residuals ${\text{e}}_{is}^{CovBat}$ are obtained by projecting the adjusted scores into observational space, via ${\text{e}}_{is}^{CovBat}=\overset{K}{\underset{k\;=\;1}{\sum }}\xi_{isk}^{CovBat}$${\hat{\phi }}_{k}+\overset{q}{\underset{l\;=\;K\;+\;1}{\sum }}\xi_{isk}{\hat{\phi }}_{l}$. Finally, the intercepts and covariate effects from the main ComBat step are added to obtain the final CovBat-adjusted observations:


$$\begin{array}{l} y_{isf}^{CovBat}={\text{e}}_{is}^{CovBat}+{\hat{\alpha }}_{f}+\;{\text{x}}_{is}^{T}{\hat{\beta }}_{f} \end{array}$$

### Advantages and disadvantages

Implementations in both R and Python are available and are clearly explained, with a call syntax highly similar to ComBat and no relevant computational burden. CovBat has been validated in one main report so far (35): here, the authors explored the effect of covariance adjustment for three different scanners on a supervised learning task directed to diagnose Alzheimer's disease from regional cortical thickness values. Second, the authors evaluated to what degree the detection of scanner effects was impaired when age and sex as covariates were preserved. In brief, CovBat outperformed ComBat regarding these tests, and in particular, the detection of sites was more strongly attenuated with CovBat. Third, four simulation scenarios (“ComBat”[no covariance site effects], “predictor affects mean” [covariance site effects], “predictor affects covariance” [covariance of site and predictor], “covariance only” [no mean or variance effects, only covariance effects of site or predictor] demonstrated that CovBat performed as well as ComBat or better–particularly when actual covariance differences were present between sites. Model transfer is implemented, so a subset of the data can be used for parameter estimation that is then applied to unseen cases (of known sites). The user can define the number of principal components (PCs) for harmonization directly, or indirectly by their explained variance. The definition of a certain number of PCs/the variance explained allows to neglect PCs that are less relevant to the overall variance explained, and thus might add unnecessary noise.

Problems with CovBat reportedly (35) arise in smaller samples (N < ~25) with many features (e.g., N ≥ 48), particularly when no covariance site effects exist–here, even an inflation of the detection of site effects after CovBat may be observed. Another limitation is that the nonlinear covariate expansion option as implemented in ComBat-GAM is not available, not allowing for more complex biological influences to be preserved.

### Longitudinal ComBat

#### Statistical foundation

Longitudinal ComBat (36) specifically addresses the issue of site effects in longitudinal data consisting of repeated measurements of the same individuals. This is accomplished by adding a time variable *t* to the model, making it time variant and allowing one to specify multiple scanning time points along with constant or time-varying covariates:


$$\begin{array}{l} Y_{isf}^{*}=\frac{Y_{isf}\left(t\right)-{\hat{\alpha }}_{f}-X\left(t\right){\hat{\beta }}_{f}-{\hat{\gamma }}_{sf}-{\hat{\eta }}_{if}}{{\hat{\delta }}_{sf}}+{\hat{\alpha }}_{f}+X\left(t\right){\hat{\beta }}_{f}+\;{\hat{\eta }}_{if} \end{array}$$

In addition to the parameter estimates for ${\hat{\alpha }}_{f}$, ${\hat{\gamma }}_{sf}$, ${\hat{\beta }}_{f}$ and ${\hat{\delta }}_{sf}$, longitudinal ComBat also models the dependence of multiple within-subject observations originating from repeated scanning of the subjects. These are modeled by a random subject-specific intercept ${\hat{\eta }}_{if}\;\sim \;N\left(0,\rho_{f}^{2}\right)$ from the feature mean estimate at baseline ${\hat{\alpha }}_{f}$ in a linear mixed model.

Similar to cross-sectional (traditional) ComBat, longitudinal ComBat can operate in two modes: in REML (restricted maximum likelihood estimator) mode, in which the repeated within-subject variance $\rho_{f}^{2}$ and the pooled error variance $\sigma_{f}^{2}$ are estimated using the restricted maximum likelihood estimator, or in MSR (mean square residual) mode, in which the pooled variance is estimated from the data, similarly to the *mean only* mode of traditional ComBat [(32, 33) Johnson et al.]. From a simulation study, Beer et al., (36) concluded that both modes of operation result in smaller standard errors for longitudinal Combat compared with traditional ComBat. Even so, while the MSR mode demonstrated the highest statistical power and allowed for more clearly discernible differences between subjects (together with unharmonized data), this method also showed an inflated type I error. The REML mode, in contrast, controlled better for type I error. The authors conclude that the choice of method results in a trade-off between type I and II error.

#### Advantages and disadvantages

Longitudinal ComBat represents an extension to traditional ComBat to handle repeated measurements. The time distances from baseline can be specified and are not necessarily equidistant. Its advantages emerge when it comes to comparisons with cross-sectional ComBat: both additive and multiplicative scanner effects are controlled to a degree that scanner or site variables were not needed in the final models of longitudinal brain changes in Alzheimer's disease. Another advantage is that no overlap cohort of subjects measured on the different scanners is needed.

What might be considered a minor limitation is that the possibility of longitudinal MRI studies comprising a *within-subject* scanner change is not considered. Beer et al. (36) have investigated this issue and found no relevant difference between a data set in which all individuals were scanned on the same scanner and a data set in which the measures were obtained on different scanners. Still, in the case of very strong scanner effects the model may need refinement. The current implementation does not offer the option to define a reference site or to train a model and apply it to new subjects from trained sites.

### Variations on ComBat

The following ComBat variants represent additional variations of the ComBat principle that, however, do not substantially alter its core statistical steps:

- Distributed ComBat (d-ComBat) (49) may be useful when sites cannot share their original data sheets with a central processing site (CS), as is needed for a mega-analysis. It requires two rounds of communications between the local sites (L) and the CS. The algorithm as whole comprises the following steps: (1) The CS assigns each LS a site (scanner) code. (2) Locally, summary statistics (of all features) are calculated and sent to the CS. (3) In the CS, by using a specific matrix decomposition, a model is estimated that returns (per feature) estimates of (i) a general intercept, (ii) the site specific regression coefficients of the covariates, and (iii) site-specific shifts. (4) These values are provided to the LS that calculate the *sum of the residuals* per feature and return it to the CS. (5) This, in turn, allows the CS to calculate the pooled variance term, which is needed by the LS to (6) finalize ComBat and obtain harmonized data. In a validation analysis, comparing d-ComBat with centrally performed ComBat, practically no difference was found in the Bayes point estimates for location and scale and the regression coefficients (of a single covariate) (49). An open source software to perform these steps is available, and theoretically the principle of d-ComBat should be transferable to longitudinal ComBat, ComBat-GAM and CovBat.
- In modified Combat (M-ComBat) one *site* is chosen as reference and all other sites are adjusted to the mean and variance of this site rather than to the grand mean and pooled variance of all sites. The principle was first presented for gene expression data (52) but was soon transferred to neuroimaging (33, 55). Reasons to choose one site as a reference site may be its large sample size, or its broader coverage or higher measurement reliability of clinical and other covariates. This approach theoretically allows for including sites incrementally without continuous adjustment. The function is also part of the R and Python version of standard ComBat (32, 33).
- Bootstrap ComBat (B-ComBat) is a computationally more expensive variant of ComBat in which the parameters of the ComBat model are repeatedly estimated (with a Monte Carlo method) and eventually averaged for improved robustness. For this approach, Da-ano et al. (55) report a slight, but consistent improvement of B-ComBat in an unsupervised hierarchical clustering example.

### Neuroharmony

#### Statistical foundation

Another novel approach that builds indirectly on the ComBat approach and allows a user to adjust imaging phenotypes (in the original paper: regional volumes) from unseen T1-weighted images has been proposed by Garcia-Dias et al. (37). The principle of this approach (referred to as *Neuroharmony*) is the use of a multivariate correlation between imaging quality metrics (IQMs) (e.g., contrast-to-noise rate, signal-to-noise ratio, intensity non-uniformity index) and relative ComBat-based corrections. More specifically, supervised ML using random forests is employed to train a system that predicts the ComBat correction factors—separately for each region-of-interest (ROI)—from such a set of IQMs. For dimensionality reduction, the latter were first reduced from 68 original values generated by the validated software MRIQC (https://mriqc.readthedocs.io) to 20 principal components. With this 3-step technique (calculating IQMs, using them as predictors in the ComBat coefficient prediction system, applying the resulting coefficients to the imaging features), T1-weighted images from unseen sites can be ComBat adjusted—as long as their IQMs lie within the ranges of the training sample of the original publication.

#### Advantages and disadvantages

Overall, a set of IQMs represents a powerful surrogate marker system that captures scanner features and that is transparent and interpretable. The algorithm was described and validated in 2020 (37), and the code written in Python is available, but has not been used in other studies so far. A clear advantage of Neuroharmony is that features from *unseen* T1-WIs from *unseen sites* can be harmonized with an existing data pool on which the random forest algorithm was trained. This is certainly a valuable function as it theoretically allows for the incremental build-up of samples on the basis of a representative starting basic multi-site dataset. Another advantage is that the tool warns the user if the IQMs of a new image lie outside the range of the training cases.

Still, there are disadvantages at the current stage: first, the IQMs need to be calculated for each new T1-WI, which requires a certain amount of effort—yet, established software scripts support this step. Second, the original implementation uses a simple covariate system (age, sex) and a defined set of image features that may not always be suitable for new study scenarios. In the case, a complete re-training would be needed which might represent a considerable effort, and might be considered as a reduced readiness level of the tool.

### Normative modeling

Normative modeling uses percentiles or z-scores to chart the variation of one (or several) targeting variables orthogonal (normed) to the variation of one (or several) covariates. The concept is famously used in pediatric growth charts, where an infant's deviation from the normative variation in height or weight may is used to track developmental milestones.

Normative modeling can be applied to any set of variables that co-vary with each other. In neuroimaging, many studies using this approach have included age and sex as covariates with regional cortical thickness or functional connectivity as the target variables. Normative modeling aims to estimate a z-score or percentile of each individual's brain measure relative to the normative distribution of individuals with the same age and sex, thus mapping the full variation in a data set and resulting in individual deviation scores. The approach has recently been used to investigate how structural brain measures of individuals with common psychiatric disorders (56–58) deviate from the norm (i.e., a normative age curve of structural brain measures).

Even though normative modeling is not a dedicated site correction tool in itself, it can be effectively used to correct for site effects in neuroimaging studies. The appeal of the use of normative modeling for site effect correction lies in the fact that the resulting z-scores derived from a normative model can be considered “normalized” for the respective covariates (e.g., age and sex). In a multi-site dataset, site can be included as an additional covariate or handled in a multilevel modeling framework [e.g., (38, 40)]. If site is used as a (factorial) covariate, the resulting z-scores describe the remaining variance in the brain metric as a distribution that is orthogonal and thus not influenced by site, enabling the comparison between z-scores across sites. In this way, normative modeling represents a different approach compared with the ComBat family. Instead of harmonizing for site by removing site effects, normative modeling models site variance as part of the normative model. In addition, the separation of variances into *aleatoric* (i.e., variation related to inter-individual differences) and *epistemic* variance (noise or modeling uncertainty, see below) allows for an uncertainty of the individual prediction and of the site effect.

#### Statistical foundation

Several approaches can be used for normative modeling–for an overview see Marquand et al. (53). Ideally, the algorithm of choice should be able to model normative centiles continuously as a function of the covariates, estimate them across the full spectrum of the centiles with appropriate precision (taking into account the varying ratio of aleatoric and epistemic variance at sparse and more dense parts of the data) and have the ability to estimate deviations for individuals *via* analytical formulae (e.g., Z-scores) (59). So far, normative modeling approaches have used hierarchical linear models, polynomial regression, Bayesian linear regression (60), quantile regression (61), generalized additive models for location, scale and shape (GAMLSS) (60, 62), support vector regression and Gaussian process regression to create centiles for normative models (53). The version that is considered in more detail here uses a hierarchical Bayesian regression approach to address the site effect problem in multi-site neuroimaging data (38). Using the example of regional cortical thickess values, the here described normative model with site as predictor in addition to age and sex returns a distribution of individual z-scores *z*_*i*_ of deviation from the normative that are orthogonal to the site factor.

Normative modeling involves estimating a z-statistic for each feature independently, which under Gaussian assumptions can be computed using the formula below, where the feature index *f* will be dropped:


$$\begin{array}{l} z_{i}=\frac{y_{i}\;-\;\mu_{i}}{\sqrt{\sigma^{2}\;+\;\sigma_{*}^{2}}} \end{array}$$

Here, μ_*i*_ is the predictive mean from a (possibly nonlinear) function mapping the the noise-free prediction for each individual sample *i* based on the specified norm, σ^2^ is the noise variance and $\sigma_{{\;}^{*}}^{2}$ is the variance of the predictions. This separation of variances is one of the key differences relative to all the other methods and allows one to make a statement on the uncertainty of the prediction per individual. Zooming in on μ_*i*_ in turn, we see that this variable is described as a combination of a 1 × *p* vector β_*j*_ containing weights associated biological variance components and a 1 × *q* vector *u*_*s*_ containing weights related to site variation.


$$\begin{array}{l} \mu_{i}=\overset{p}{\underset{f=1}{\sum }}x_{ij}\beta_{j}+\overset{q}{\underset{b=1}{\sum }}z_{is}{û}_{s}+\;\gamma_{i} \end{array}$$

γ_*i*_ is an optional component in order to add non-linearity, for example via a Gaussian process or B-splines. Finally, this noise free prediction is linked to the noisy observation via:


$$\begin{array}{l} y_{i}=\mu \left(x_{i}\right)+\;\epsilon_{i}\\ Y=\mu +\epsilon ,\;with\;\epsilon \sim N\left(0,\;\sigma^{2}\right) \end{array}$$

The model in our recently specified implementation adds hierarchy by placing shared priors and hyperpriors θ_0_ over both variance components: $u\sim N\left(0,\Sigma_{j}\right)$ and *u*_*s*_ ~ *Inv*Γ(2, 2). All free parameters, summarized in θ are estimated by performing Bayesian inference:


$$\begin{array}{l} p\left(\theta |X,y,\theta_{0}\right)=\frac{p\left(\theta ,X,y,\theta_{0}\right)}{p\left(X,\;y,\theta_{0}\right)}=\frac{p\left(\theta |X,y,\theta_{0}\right)}{p\left(X,y,\theta_{0}\right)} \end{array}$$

#### Advantages and disadvantages

Normative modeling has several advantages, the most important of them being that it does not remove any biological variance of interest that might be confounded with site variance. This characteristic makes normative modeling also applicable in a setting when biological information is not uniformly distributed across sites (for example, more female or more control group individuals in one site than the other). Rather, NM results in direct predictions of the mean and the variance for the observed distribution, allowing to calibrate data to population centiles. This step places all data in a common reference space, whilst retaining the ability to relate these centiles back to the original scale of the data (e.g., *via* site-specific means and variances). The return of predictive mean and variance also allows to validate the predictions of NM, and thus the goodness of the subsequently calculated z-scores (see general discussion for details).

A further advantage of NM is the hierarchical structure within the Bayesian framework, which permits the application and generalization to new sites and data points. For example, it allows for or the re-use of trained posteriors as priors to inform further estimates. This way, the normative model can also be used to calibrate predictions for unseen sites, as demonstrated in Kia et al. (39).

Beyond that, recent extensions of the normative modeling approach variants of the framework can model no-Gaussian effects *via* likelihood warping (60), and GAMLSS (62). Kia et al. (40) developed a variant in which, once trained, a normative model can be applied to decentralized neuroimaging data, similar to decentralized Combat.

One practical disadvantage of normative modeling—yet, depending on the non-linear algorithm of choice–is the computational cost and complexity of the model. Gaussian progress regression, for example, can become very time and memory expensive with growing *n* (*O*(*n*^3^)) [see also (60)]. This may limit its current use to low dimensional imaging data with a few hundred features per subject but not voxel or vertex wise maps or connectomics data that may contain over 10^6^ features. Further, if covariates are not well-selected and show no association with the imaging phenotype, this may lead to the model failing to predict as there is no variance captured by the model.

### Adjustment of raw imaging data for the purpose of site harmonization by deep learning methods

Deep learning methods aim to harmonize raw images by using image translation techniques rather than correcting secondary features extracted from the post-processed images. These can be classified as supervised methods, which typically require traveling subjects and must be planned prospectively (63) and in unsupervised methods, such as variational auto-encoders (41) or CycleGAN (64) where MR images are often separated into well-defined domains in terms of scanners or sites.

#### Statistical foundation

Several deep learning methods for site correction are based on generative adversarial networks (GANs) (65), that have achieved remarkable results in creating, adjusting, and enhancing images for artistic as well as scientific applications. In one formulation of adversarial learning, the aim would be to extract a set of predictive features from the image that are maximally predictive of a specific outcome (e.g., classifying Alzheimer's disease, or any other diagnosis) and at the same time maximally uninformative of the site or scanner where the data originated. This “minimax game” has inspired a game-theoretic formulation, and GANs combine and train two neural networks: one (the generator) extracts predictive features from the input data, while the other (the adversary) tries to distinguish which site the data came from.

The VAE-GAN method (41) uses a variational autoencoder to embed the predictive features into a latent space, such that the latent space features are useful for the main task (predicting diagnosis), but defeat the adversary that tries to predict the site where the data originated. Practically, this is achieved by adding a “gradient reversal layer” to shift neural network parameters to defeat the adversary. When trained successfully, the VAE produces a so-called scanner-invariant set of predictive features–features that do not depend on the site of origin of the data. A third penalty term, which is optional, and can also be simultaneously optimized, can be used to reconstruct the original image from the latent space as accurately as possible. As noted below, in StyleGAN extensions of this basic approach, the latent space data can also be combined with a site code, yielding corrected images that match data from a certain reference site.

Zhao et al. (66) noted that this same adversarial principle could also be used to de-confound deep learning models for other biological variables potentially co-varying with site, such as age and sex. They argue that this type of adversarial site correction is crucial to avoid training DL methods that incorrectly learn accidental features of a cohort, and then fail badly when applied to new datasets [the “domain shift” problem in ML (8)]. Dinsdale et al. (67) provide a practical implementation of this set-up for a brain age prediction task, using multiple adversaries to adjust for multiple confounds (including site and sex).

#### Advantages and disadvantages

In practice, adversarial deep learning methods are prone to overcorrection when sites have used different scanning parameters, and are also confounded by demographic and clinical differences, such as clinical diagnoses, age ranges, or ethnicity, which might lead to site corrections that remove critical biological differences. In these situations, the demographic and clinical conditions need to be strictly controlled and matched.

Recently, deep learning methods have successfully achieved diverse image translations by disentangling the image into “content” and “style” spaces (68), where contents represent low level information in images such as contours and orientations, and styles may be considered as including high level information such as colors and textures. Based on this distinction, several methods have been proposed. Dewey et al. (43), for example, used this breakdown (including the scanning of a small overlap cohort) to show promising results for harmonization of T1-weighted images from different scanners and protocols: indeed, the consistency of segmentation results was significantly improved. As an extension of Dewey's work (43), a recent MRI harmonization method, called CALAMITI (Contrast Anatomy Learning and Analysis for MR Intensity Translation and Integration) (69), relies on intra-site supervised image-to-image translation and unsupervised domain adaptation for multi-site harmonization. This requires training a disentangled representation model with intra-site multi-contrast images (T1- and T2-weighted images) of the same subjects and retraining the model for a new site *via* domain adaptation–no sample population imaged across sites is needed.

Unlike CALAMITI, several other methods require only images from a single contrast and can learn multi-site harmonization simultaneously. For example, DRIT++ (70) embeds images in a site-invariant content space capturing information shared across sites and a site-specific style space for every pair of sites. The encoded content features extracted from an image of one site are combined with style features from another site to synthesize the corresponding harmonized image. However, DRIT++ cannot fully utilize the entire training dataset and can only learn from two sites at a time, causing it to miss global features that can be learned from images of all sites. Failure to fully utilize training data likely limits the quality of the generated images.

**Figure 3:**
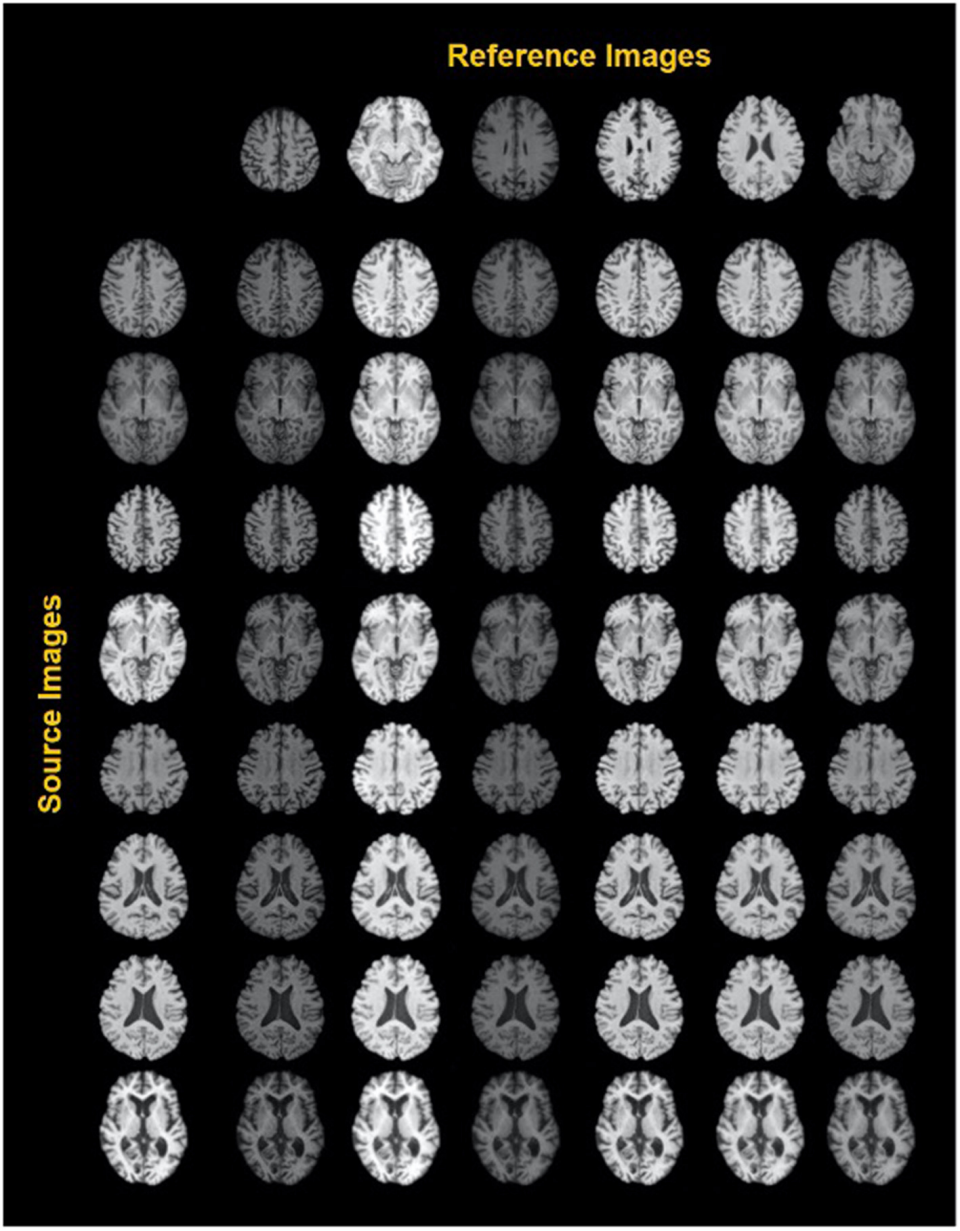
Exemplary results of a style-encoding GAN. First row shows six reference images (columns 2–7) differing in their contrasts between gray matter, white matter, CSF and background, and the first column showing different source images at different axial slice positions. Harmonized images in each row demonstrate well-maintained anatomical structures and at the same an alignment of the contrast features to the reference column. See Liu et al. (44) for details. Reproduced with permission.

A similar study by Liu et al. (44) was carried out to harmonize MRI images from multiple arbitrary sites using a style transferable GAN by jointly considering images from all sites. The approach conceptualizes image harmonization as a pure style transfer problem rather than a domain transfer problem: the appearance of the harmonized image is determined by the style features extracted from the reference image. Based on a large, diverse training dataset, the model proved capable of being applied to unseen images (see also Figure 3).

More recently, MURD (71) enforces explicit disentanglement of content and style features, allowing it to produce harmonized images with diverse appearances and significantly better preservation of anatomical details. Disentanglement safeguards harmonization against altering anatomical image contents and allows gradual and controllable harmonization through interpolation of style features. Other than that, the harmonization target is not specified by a single reference image, as suggested in Liu et al. (44), but rather by a site label, which determines the output branch of the style generator and the style encoder. A latent code sampled from the standard Gaussian distribution then determines an appearance specific to the site.

## Discussion

The dilemma in mitigating site effects in multi-site neuroimaging studies is illustrated in Figure 1: on one hand, sites differ in acquisition factors that reduce the statistical power to detect the correlations of interest. On the other hand, sites often also reflect different clinical and neurobiological characteristics, or in an extreme case, clinical or neurobiological characteristics that are unique to a site. If these factors influence the imaging measure, their contribution to the phenotypic variance should thus not be removed (“overcorrection”). In turn, “undercorrection” of the site effect diminishes the power of subsequent regression analyses and leads to poor generalization in supervised machine learning or to site factors dominating subgroup formation in unsupervised learning, such as clustering. All discussed methods thus aim to optimize the power of a pooled analysis by “emulating” a situation in which all subjects were scanned on the same imaging platform.

### Validation of ComBat family methods

Regarding its basic validation in neuroimaging, Fortin et al. (32) compared ComBat with four other correction techniques, analyzing if unwanted variation induced by the scanner/acquisition protocol was removed and if between-subject biological variability was preserved. For the latter, an empirical validation scheme was set up, defining a pool of voxel-wise diffusion scalars (e.g., fractional anisotropy) associating with age vs. another pool not associating with age. Within this scheme (which might be referred to as “silver standard” as it is based on external knowledge, for example biological aging assumptions, and not a neutral statistical metric), ComBat proved superior to (1) global scaling (in which one global shift and one scale parameters is estimated for each site), (2) functional normalization (that models the variation quantile functions as a function of site), (3) RAVEL (that estimates latent factors from a control region in the brain maps), and (4) surrogate variable analysis (that uses PCA to remove automatically selected components). Fortin et al. (33) extended validation work to cortical thickness measurements, showing that site could not be predicted by a support vector machine after harmonization and that age prediction by linear regression and multivariate methods was not impaired by ComBat (but also not improved). Expectedly, the combination of *unbalanced* samples regarding the covariate distribution and a method not considering the covariate system (such as *unadjusted residualization*) led to removal of age-related signal, as shown by other work (33, 72). Using simulations, Orlhac et al. (48) studied the effect of ComBat on site-by-covariate interactions, concluding that ComBat preserved covariate effects where present in specific sites without introducing spurious covariate effects where no such effect was present. To further refine ComBat, Zhang et al. (73) suggested first analyzing the actual deviation of moments (mean, variance, skewness, kurtosis) from a reference site and then performing only the adjustments needed.

How does ComBat relate to meta-analysis and mega-analysis? Mega-analysis is known to provide larger power compared with meta-analysis (74), and one additional argument for mega-analysis is its potential to perform analyses across a larger spectrum and richer variability of covariates, including interaction analyses. Thus, a practical approach to this question was to compare a mega-analysis on ComBat-harmonized data with a standard random effects (RE) meta-analysis and with a mixed effects (ME) mega-analysis where site was modeled as a random effect (51). Accepting disease effects (schizophrenia vs. controls) as a silver standard for which maximization is sought, stronger group effects were detected for ComBat compared with RE meta-analysis, and ComBat also offered a less strong but significant advantage over ME mega-analysis. From this perspective, d-ComBat is an interesting addition as it combines the privacy-conserving aspect of meta-analysis with the possibility to harmonize the data (49). Here, while a gain for meta-analysis is expected, a formal comparison of d-ComBat harmonized meta-analysis against classical meta-analysis is still lacking.

ComBat-GAM extends ComBat by a flexible extension to nonlinear covariate effects. After it was shown to preserve lifespan aging trajectories (11), more validation papers have followed, one showing an advantage (stronger patient|control differences and stronger age-related declines) compared with linear ME mega-analysis that modeled a site random effect and age-related random slopes (75). Simulation experiments have further corroborated the robustness of ComBat-GAM (11) with two practical implications: first, sites with partially overlapping ranges of the covariate of interest can be better harmonized compared with sites that show disjoint ranges. Second, a large total number of subjects seems to outweigh the necessity for balanced samples (see also Box 1). Yet, it should be added that the validation of ComBat-GAM has not been very strict in that (1) ComBat only had a quadratic but no higher order terms and (2) the effect of ComBat-GAM on subsequent MVPA was not compared with the quadratic ComBat.

Box 1 **Caveats and preliminary conclusions for the ComBat family.**

**Table:** 

**Sample size**	The use of Empirical Bayes can improve the estimation and removal of site effects in datasets even with small sample sizes of at least 20–30 subjects (32, 76), yet, samples with less than 20 subjects might overstrain the algorithm and lead to unreliable priors and hyperparameters. The problem might be aggravated when covariates are added that further compartmentalize the data.
**Dimensionality of features**	Generally, the computational burden of ComBat is low. Both extracted features in the magnitude of dozens and hundreds up to voxel-wise measures can be entered to the ComBat implementation. For some implementations, 3D files need to be rewritten as [N,1] vectors. ComBat cannot be run for a single feature (see above).
**Balanced sample sizes**	As pointed out above, a larger total sample size seems to outweigh the degree of balance which might serve as a hint toward a rather inclusive strategy.
**Distribution of covariates**	As site effects and covariate effects across all sites compete with each other in the ComBat model, it is recommended that the distribution of covariates are not disjunct but overlap between sites (11, 33).
**Separate handling of different types of features**	It may be critical to combine subsets of features with a diverse range (and different units) (for example, combining cortical thickness [range 1-5 mm] and subcortical volumes [20–100 mm^3^]) in one dataset for ComBat. This may disturb the standardization step that is based on the pooled variance across sites and all features. It is thus recommended to harmonize these distinct feature subsets separately, which also preserves the interpretability of the position and units of the ComBat adjusted values.
**Expected non-linear covariate effects**	ComBat-GAM might be considered the most flexible tool when the envelope of the non-linearity is entirely unknown. For life-span studies, its primary validation paper (11) thus represents a guideline. For other studies, ComBat with a set of pre-defined non-linear extensions might be comparably suitable.
**Additional harmonization of covariance**	This newer extension is worthy of consideration under certain pre-conditions (Chen et al.; 11): (1) Data exploration demonstrates that covariance actually differs between sites (scanners), (2) sample sizes are sufficiently large to provide reliable estimates for the covariance, (3) results should be compared to conservative standard ComBat to understand the impact of the additional step.
**ComBat in scenarios without full access to subject-level features**	Here, distributed ComBat might serve as a workaround to rescue power that is lost in classical RE meta-analysis.
**Model transfer to unseen cases of known/unknown sites**	Unseen cases of sites known to the model can be corrected by ComBat, ComBat-GAM or CovBat. The transfer of the corrective model to data from unseen sites is not directly supported by the ComBat family of methods - only through additional adaptations (e.g., Neuroharmony (37). Also see *discussion*.

Longitudinal ComBat (36) is relevant for serial multi-site imaging studies: In the ADNI sample, an advantage over cross-sectional ComBat was demonstrated regarding the attenuation of scanner effects and the power to detect diagnosis-by-time effects. An alternative for the management of longitudinal (structural MRI) studies is to use tools that combine the serial raw images to derive a *differential* result and then correct this by using standard ComBat. Examples for such approaches are deformation based morphometry (77), edge-displacement based approaches to measure atrophy [https://fsl.fmrib.ox.ac.uk/fsl/fslwiki/SIENA] or specific implementations of FreeSurfer for longitudinal studies (78).

More recently it has been shown that ComBat leaves covariance patterns in the residuals that might still contain scanner effects and obscure biomarker effects (35). For this, CovBat harmonizes the covariance of the residuals after standard ComBat. Yet, *structural* covariance is not only a confound, but contains important network information (79) and such across-subjects covariance is also present in task-fMRI (“co-activation networks”) and structural and functional connectomics. Overall, CovBat could be useful for large datasets in which strong site-related covariance patterns exist and in which a high proportion of observations over features is available.

Despite these extensions of ComBat to incorporate non-linear, longitudinal and covariate site effects, all variants of the ComBat family rely on principal assumptions that limit the application of its use. First, the ComBat algorithm requires the noise term, ϵ_*isf*_ (the residuals) to be at random (*Gaussianity*). At this point, no ComBat variant can accommodate for skewed noise distributions, which have, however, been found to be present in certain types of neuroimaging data (60). Secondly, the variance, including the noise, has to be equal and independent from the predictor (*Homoscedasticity*). ComBat should not be used in cases where those two assumptions are violated.

Beyond that, the differentiation between “wanted” (biological) and “unwanted” (site-related) variation is not usually clear cut. In contrast, the modeler is often confronted with the problem that variance that is fully shared between site and variables of interest cannot be uniquely attributed to either. In this case one could argue that it is safest to discard this information. This is an indirect critique toward the ComBat method, for which it has been argued that the step of re-adding part of the signal (the “wanted variance confounded with site”) leads to overconfident results due to a relative shrinkage of the noise term (46). Thus, despite the loss of sensitivity in case site is correlated with effects of interest, it could be argued that given the risk of the artificially deflated residuals and the consequently (sometimes severely) inflated Type I error, the safest way to use ComBat might be to disregard of the estimates *Xβ* instead of adding them back in.

### The use of ComBat in MVPA applications

Most ComBat studies have evaluated its effects in MVPA such as multivariate age estimation (11, 32, 33). ComBat as a preparation step for MVPA has been the focus of a comparative study in onco-radiology (55): Three different ComBat variants were run before three supervised ML algorithms (multivariate regression with LASSO, random forest, support vector machine). Generally, ComBat harmonization improved MVPA performance at the level of several performance measures. Still, these improvements need a closer investigation regarding their generalizability as too much variance might be removed by ComBat which may lead to overconfident estimates further down the processing. This more general problem of a residualisation approach is even obvious in a two factor (site, group) ANOVA (46). Similarly, CovBat might remove biologically relevant covariance confounded with site: For example, neurodegeneration of a certain severity disrupts the physiological network organization visible as structural covariance (80). By this, a site with an imbalanced sample (e.g., more patients) could be falsely cleaned of relevant information.

Box 1 gives an overview over caveats and preliminary conclusions for the ComBat family.

### Normative modeling to control for site effects

Normative modeling (NM) provides an alternative approach: NM aims to *map* the full variability of a feature across the population in the form of percentile scores or z-scores onto a set of predictors (including site), resulting in individual prediction, along with a measure of uncertainty per individual and per site. Conceptually, it may be understood as a *normalization* of brain measures per site instead of estimating a site effect and removing it. Several methods have been used to achieve this normalization (or mapping) step–for an overview see Marquand et al. (53). The approach we discussed in this paper makes use of hierarchical Bayesian regression (HBR), modeling sites by placing a shared prior over site, and allowing for non-linearity by adding a Gaussian process component. This effectively pools estimates across *sites*, which is a different approach to modeling site variation than pooling across voxels or regions of interest, as is done by ComBat and other harmonization techniques. The differentiation between subject-related (aleatoric) variability and noise related (epistemic) variability allows for an estimate of uncertainty of the individual prediction, with “prediction” denoting the prediction of the z-score of one individual per feature and the corresponding underlying variance. In other words, epistemic variance (noise) can be reduced by adding more data, whereas aleatoric (subject related) variance cannot be reduced. The z-score may be interpreted as a corrected value of this feature, considering site and other covariates included in the model. The fusion of the two different variance types in calculating the z-score also means that the amount of uncertainty in the model that can be attributed to data sparsity (epistemic noise) is included in the z-score and can be tracked back. The difference from other methods of calculating a z-score lies in the differentiation of variances, which results in a z-score per individual that does not only map onto an individualized mean, but also to an individualized variance, as not only the mean, but also the variance varies with the predictor. NM has been used in various neuroimaging applications and is conceptually universal and not specific to certain imaging modalities or features (53, 60). Yet, its use for site effect correction is fairly new. NM is similar to ComBat-GAM in terms of the flexible modeling of non-linear covariate effects although it can also accommodate non-Gaussian distributions. Other than in ComBat that uses variance across features in the EB step, in NM the z-scores are independently estimated for each feature. The exact derivation of percentile scores for NM can be computationally demanding (although less demanding approximations exist, see (53, 60) which might lead to limitations in use in the case of large size features (see also Box 2).

Box 2 **Caveats and preliminary conclusions for normative modeling.**

**Table:** 

**Sample size**	Although the separation of variances in the NM allows one to quantify the sparsity of data points along the predictor, the training of a normative model requires a relatively large sample size to adequately map the variance (300–500 individuals, more is better). A normative model can also be applied at the level of a single individual.
**Dimensionality of features**	In the current implementation of (38) NM is applied unit-wise (feature, voxel). This assumes that features are independent from each other and do not share variance. 3D files need to be rewritten as [N,1] vectors. NM can be applied to large data sets with many features, but requires a massively parallel computational approach.
**Balanced sample sizes**	The separation of variances in NM allows for an estimate of uncertainty based on the density along the covariate. This prevents overconfident estimates in the case of unbalanced samples.
**Distribution of covariates**	Covariates do not necessarily need to overlap (but this is recommended). Missing parts in covariate distributions may be filled in/estimated via prior distributions, but estimates from priors will always be more noisy than from actual data.
**Separate handling of different types of features**	Different types of features or feature sets can be processed in one run if adequate computational power is available (also see above).
**Expected non-linear covariate effects**	Normative modeling with a Gaussian process, or a GAM addition, allows for non-linear covariate effects (not only with respect to the mean, but also the variance of the prediction).
**Additional harmonization of covariance**	*Not considered*.
**NM in scenarios without full access to subject-level features**	To perform NM, full access to the raw data is needed.
**Model transfer to unseen cases of known/unknown sites**	*See discussion*.

A direct comparison of NM and ComBat is difficult as the output of the two methods results in values on different scales (z-scores vs. adjusted data in original space): Whereas Combat subtracts an estimate of the site effect from the data, NM accounts for the site effect variation in the standard deviation that the z-scores are based on. Bayer et al. (38) used a special pipeline to make the two measures comparable: Z-scores were calculated from the ABIDE dataset by NM that entailed site (and other covariates) as predictor. The same data were adjusted using ComBat with protection of the covariates and then forwarded to NM with only the covariates (but lacking site). This enabled us to assess the accuracy of the prediction of (1) the *mean* (Pearson's correlation between predicted and observed values; standardized root mean square error) and (2) the *variance* values (explained variance, mean standardized log loss) that underlie the eventual z-values. According to all these performance metrics, the NM-based correction model with site effect included performed better. This procedure, at the same time, highlights the downside of ComBat that (for itself) does not allow for a direct way to validate the truthfulness of its adjustments: this information could only be retrieved by appending the NM step. This problem is present both when data are projected to an artificial average site or a reference site, and it also hampers the comparison between independent ComBat procedures. In addition, Bayer et al. (38) demonstrated that ComBat, even when retaining covariates, led to a poor calibration of the predictions reflected in excessive loss of the original variance. Interestingly, sites could still be predicted from the NM z-values after the ComBat correction, a sign of undercorrection. One underlying reason might be the underestimation of the site-by-covariate interactions in ComBat, which are better captured by the more variance conserving normative modeling approach. This preservation of variance in NM also makes the resulting z-statistics back-translatable and allows for an intuitive understanding of deviations in the original scale of the data.

Another difference to ComBat is the flexibility that is inherent in the distribution based approach of the NM framework. Fraza et al. (60) suggested that white matter microstructure measures (such as mean diffusivity, fractional anisotropy, radial diffusivity, and axial diffusivity) derived from DTI data may deviate from Gaussianity, making the ComBat approach not applicable. They further showed how a likelihood warping extension to a Hierarchical Bayesian Regression normative model based on B-splines was able successfully model skewed distributions. Work by Dinga (62) using generalized additive models of location, scale and shape supported this result, and also showed explicitly how NM can be used in the case of non-linear, heteroscedastic and non-Gaussian data.

### Transfer of site correction models to unseen data

“Unseen data” may refer to unseen subjects from known sites, or data from totally new (unseen) sites. As ComBat outputs average levels for each feature, covariate effects, and site-specific position and scaling estimates, the model can be applied to unseen subjects' data from known sites. Technically, this option is implemented for traditional ComBat, CovBat and ComBat-GAM. Da-ano et al. (81) investigated if transferring ComBat to an unseen random split subsample of the data would compromise supervised ML; in brief, no relevant differences were found compared to ComBat applied to the full sample. Yet, the study dealt with tumor radiology and nuclear medicine markers and results are likely not generalizable to neuroimaging due to sample sizes, covariate effects and measurement reliability. Intuitively, the transfer to new subjects from known sites may work more robustly if the biological covariates of interest between the training and test subsamples are balanced. Such balance should also help MVPA studies to attribute the overall loss of performance to (i) the *site effect model* vs. (ii) the generalizability of the actual *multivariate prediction model*.

The situation is more difficult if a site effect correction needs to be applied to data from completely unseen sites. This, however, is a requirement for fully transferable diagnostic MVPA pipelines that do not require calibration or training. One solution is to define a reference site for ComBat that allows for a build-up of adjusted data—yet, the properties of such a reference site cannot be easily formalized and depend on the context. Two-stage systems are conceivable in which a very large multi-site sample is harmonized first, and these harmonized data are then used as a stable (2nd level) reference site for new sites. Overall, so far, ComBat cannot correct for site effects of data from unseen sites—which is a limitation regarding MVPA.

*Neuroharmony* (37) uses the multivariate correlation between basic image quality metrics (IQMs) and ComBat coefficients that can be learned by machine learning (elastic net). For data from unseen sites, these same IQMs are then calculated and used to predict the necessary ComBat coefficients by the formerly trained elastic net. While the principle is elegant and could serve as template for the ComBat family and other image modalities, it needs retraining for different features (as compared to the initial publication) and also for other covariate schemes. This customizing step may hamper its wider use.

In the normative modeling context, transfer to unseen sites can be done in three ways: first, a trained model can be used as a normative reference model for individuals from new sites. This approach has been successfully demonstrated in Kia et al. (39) who trained a normative model based on Hierarchical Bayesian Regression in Python from the PNC toolkit on four different sites and subsequently created predictions for three new sites. Kia et al. (39) showed that the HBR approach with a calibration step was able to make adequate predictions for the mean and variance of the new sites, as indicated by performance measures such as root mean squared error, mean standardized log loss and Pearson Correlation Coefficient, and reliably revealed cortical thickness differences between individuals from different clinical cohorts. Second, the posterior of a trained model can be used to inform a prior when training a model on unseen data. This option might be useful when the new sites are small, as the informed prior prevents overfitting. Thirdly, the posterior can be used as a prior distribution to make predictions without informing it with the new data. The idea behind this approach is that a posterior from training some sites might be (at least to some degree) informative for the range of variation in new sites. Indeed, this approach has been shown to make only good predictions for the mean, but perform less well for the variance of the new normative distribution (39).

### Sharing (pooling) of information across features

The question whether sharing or pooling of information across features is a benefit in site effect correction methods is an ongoing discussion. Combat uses a shared variance component at feature level during the Empirical Bayes step by borrowing information from other features that as a whole hold more useful information than the single, potentially noisy, feature. In principle, the same logic still applies to less noisy features (for example regional cortical thickness values each of which is based on hundreds of vertex points). NM, on the other hand, deliberately treats features as independent, not making any specific assumptions about the relatedness of the features. What remains unconsidered in *both* ComBat and NM is the spatially structured smoothness of the data with a higher similarity of anatomically neighboring voxels. This could be a further developmental step, and particularly hierarchical Bayesian approaches have been successfully used with functional MRI to incorporate temporal and spatial dependencies (82).

### Deep learning applied to multi-site imaging studies

**Figure 4:**
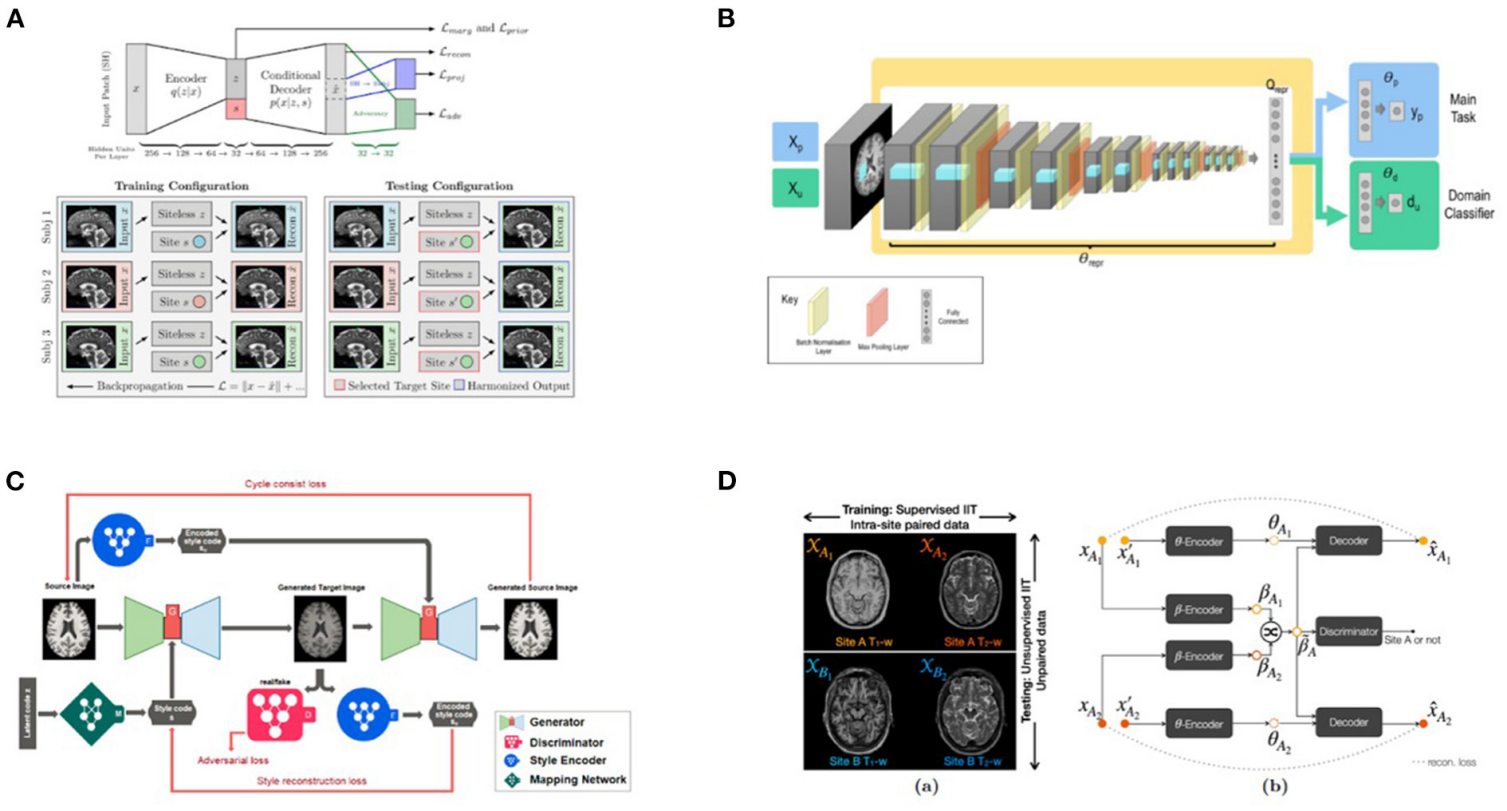
Four GAN-based deep learning harmonization models. **(A)** The network architecture in Moyer et al. (41). The invariant representations to and from the images are learned using an encoder/decoder architecture, with a one-hot vector to represent the protocol identifiers. **(B)** General network architecture in (67). The network is formed of three sections: the feature extractor with parameters, the label predictor with parameters, and the domain classifier with parameters. The domain invariant features (from the feature extractor), which are used in domain-invariant label predictions. are learned by confusing the domain classifier. The represents the input data used to train the main task with labels, and represents the input data used to train the steps involved in unlearning scanner with labels d. **(C)** The architecture of the style-encoding GAN (44). Generators learn to generate images by inputting a source image and a style code. The anatomy of the brain MRI was preserved using a cycle-GAN architecture, and the harmonization was achieved by inserting a style code into the images. Reproduced with permission. **(D)** (a) Given T1-w and T2-w images from Sites A and B, the method from Zuo et al. (69) learns the site-invariant anatomy from supervised image-to-image translation (T1–T2 synthesis) and site-variant contrast from unsupervised image-to-image translation (harmonization), where is learned to control the image contrast after harmonization, and is learned to preserve the anatomical information.

Generative adversarial networks in different conceptual realizations (see Figure 4) allow for a separation between features predictive of the desired outcome and features that are attributable to the sites. By accessing the latent space features, an image of one site can then be transformed to an equivalent of another (reference) site. The site|covariate confounding issue remains, however, and recent extensions of the GAN principle thus direct additional adversarial networks to covariate effects to protect the model from losing individual biological information. When combined with sufficiently large and diverse training samples, the GAN principle could eventually allow for adapting the appearance of data from an unseen site to a reference style which opens the perspective toward highly powered pooled studies. Currently, these developments focus on macroanatomical and diffusion MRI, and more validation studies are needed on the risk of overcorrection and on their effects on post-processing pipelines. For this, robust silver-standards need to be defined that prove that neurobiological and clinical information content is not corrupted.

### When to correct for site effects

Opportunities to correct for site effects arise at different stages of the processing pipeline, and they also arise at different stages of data collection. For example, different scanner vendors and acquisition protocols will result in different raw brain images. Later, researchers at different research centers might use different software products to analyze those images, on different operating systems. Once the data are uploaded to a public server, the meta- or mega-data analyst will already be faced with a complex interaction between those scanner effects and other variables that differ between scanner locations (disease status, sex, age). Naturally, a harmonization of site effects at a stage as early as possible, including coordinated data collection, and at every step of the pipeline along the way would be ideal. Still, in retrospective harmonized data analyses that are predominant in ENIGMA and other collaborative research initiatives with public data sets, the researcher may only have access to measures derived from the original image, making it less feasible to correct for site effects at an earlier stage. We recommend integrating correction methods as soon as possible in the pipeline once the user has access to the data to prevent confounded downstream analyses. For this reason, we also highlight deep learning methods—some of which operate on raw MR images.

### Prospective site effect correcting methods

We refer the reader to recent studies [i.e., (63, 83)] for a discussion and comparison of the benefits of the use of prospective site effect prevention methods, such as the use of phantom scans or the 'traveling subjects' approach, although we have not focused on it here, for the following reasons.

To begin with, the target audience for this manuscript is researchers who plan to work with open, public or consortium data. This type of data is usually only accessible and being pooled after data collection has been concluded—and sometimes after several pre-processing steps have already been taken—meaning that the user has little or no control over those steps. Thus, we prioritized methods and strategies that can be applied after the data has already been collected and minimally pre-processed.

Secondly, the origin of site effects is heterogeneous and not necessarily independent of biological variation; the extent of any interaction is not always fully known. For example, they can arise from scanner related factors, such as scanner platform and sequence details, which could potentially be alleviated using phantom scans. Yet, other factors affecting the postprocessing (software version, processing pipeline and operating system) equally introduce biases, and even QC procedures deserve a harmonization step. Given this complexity and the fact that some image modality specific intermediate steps are needed to transform the phantom or traveling subject information into an effective site correction, we refer the reader to another overview paper on this matter (see introduction of deep learning Section Adjustment of raw imaging data for the purpose of site harmonization by deep learning methods).

## Conclusions

For multi-site neuroimaging analyses, different methods to attenuate site effects have become available, comprising different statistical principles, degrees of use, level of validation, and readiness level. Well-founded approaches comprise the ComBat family and, more recently, normative modeling that by its flexible concept is increasingly adapted for site effect correction. Newer developments such as adjusting raw multisite MRI data through deep learning techniques show promise, but call for more accessible implementations and validation. We encourage the open sharing of site effect correction software and a detailed explanation in the methods sections of future manuscripts. We encourage the open sharing of site effect correction software and a detailed explanation in the methods sections of future manuscripts. Given that a chosen site adjustment method can significantly influence the outcome of the final analysis, future studies comparing results from multiple site correction methods could improve the replication and generalizability of large-scale, multisite neuroimaging studies.

## Author contributions

JB: conceptualization, methodology, software, writing—original draft, writing—review and editing, visualization, and project administration. PT: conceptualization, methodology, writing—original draft, writing—review and editing, and funding acquisition. CC: methodology and writing—review and editing. ML: methodology, visualization, and writing—review and editing. AC, NJ, and AM: writing—review and editing. AP: visualization. LS: conceptualization, writing—review and editing, project administration, supervision, and funding acquisition. PS: conceptualization, methodology, software, writing—original draft, writing—review and editing, visualization, project administration, and supervision. All authors contributed to the article and approved the submitted version.

## Funding

PT was supported by NIH grants R01AG058854, U01AG068057, RF1AG057892, R01AG060610, R01MH116147, P41EB015922, R01MH121246, R01NS107513, R01MH123163, and U.S. Alzheimer's Association grant ZEN-20-644609. He was also supported by NIA T32AG058507 and NIH grant U54EB020403 from the Big Data to Knowledge (BD2K) Program. PT also received partial research support from Biogen, Inc. (Boston, USA) for work unrelated to the topic of this manuscript. AC was supported by the National Institute of Neurological Disorders and Stroke (grant numbers R01 NS085211 and R01 NS060910), the National Multiple Sclerosis Society (RG-1707-28586), and a seed grant from the University of Pennsylvania Center for Biomedical Image Computing and Analytics (CBICA). AM gratefully acknowledges support from the European Research Council (ERC, grant ‘MENTALPRECISION' 10100118), the Wellcome Trust under a Digital Innovator award (‘BRAINCHART,' 215698/Z/19/Z) and the Dutch Organization for Scientific Research (016.156.415). NJ was supported by NIH grants R01AG059874 and R01MH11760. LS was supported by the National Institute of Mental Health of the National Institutes of Health (R01MH117601) and by a NHMRC Career Development Fellowship (1140764).

## Conflict of interest

The authors declare that the research was conducted in the absence of any commercial or financial relationships that could be construed as a potential conflict of interest.

## Publisher's note

All claims expressed in this article are solely those of the authors and do not necessarily represent those of their affiliated organizations, or those of the publisher, the editors and the reviewers. Any product that may be evaluated in this article, or claim that may be made by its manufacturer, is not guaranteed or endorsed by the publisher.

## References

[1] Thompson PM Jahanshad N Ching CRK Salminen LE Thomopoulos SI Bright J. ENIGMA and global neuroscience: a decade of large-scale studies of the brain in health and disease across more than 40 countries. Transl Psychiatry. (2020) 10:167. 10.1016/j.biopsych.2020.02.167 32198361 PMC7083923

[2] Psaty BM O'Donnell CJ Gudnason V Lunetta KL Folsom AR Rotter JI. Cohorts for heart and aging research in genomic epidemiology (CHARGE) consortium. Circ Cardiovasc Genet. (2009) 2:73–80. 10.1161/CIRCGENETICS.108.829747 20031568 PMC2875693

[3] Habes M Pomponio R Shou H Doshi J Mamourian E Erus G. The brain chart of aging: machine-learning analytics reveals links between brain aging, white matter disease, amyloid burden, and cognition in the istaging consortium of 10,216 harmonized MR scans. Alzheimers Dement. (2021) 17:89–102. 10.1002/alz.12178 32920988 PMC7923395

[4] Bethlehem RAI Seidlitz J White SR Vogel JW Anderson KM Adamson C. Brain charts for the human lifespan. Nature. (2022) 604:525–33. 10.1038/s41586-022-04554-y 35388223 PMC9021021

[5] Rutherford S Fraza C Dinga R Kia SM Wolfers T Zabihi M. Charting brain growth and aging at high spatial precision. Elife. (2022) 11:e72904. 10.7554/eLife.72904.sa2 35101172 PMC8828052

[6] Sudlow C Gallacher J Allen N Beral V Burton P Danesh J. UK biobank: an open access resource for identifying the causes of a wide range of complex diseases of middle and old age. PLoS Med. (2015) 12:e1001779. 10.1371/journal.pmed.1001779 25826379 PMC4380465

[7] Davatzikos C Sotiras A Fan Y Habes M Erus G Rathore S. Precision diagnostics based on machine learning-derived imaging signatures. Magn Reson Imaging. (2019) 64:49. 10.1016/j.mri.2019.04.012 31071473 PMC6832825

[8] Dockès J Varoquaux G Poline J-B. Preventing dataset shift from breaking machine-learning biomarkers. Gigascience. (2021) 10:giab055. 10.1093/gigascience/giab055 34585237 PMC8478611

[9] Varoquaux G. Cross-validation failure: small sample sizes lead to large error bars. Neuroimage. (2018) 180:68–77. 10.1016/j.neuroimage.2017.06.061 28655633

[10] Wolfers T Buitelaar JK Beckmann CF Franke B Marquand AF. From estimating activation locality to predicting disorder: a review of pattern recognition for neuroimaging-based psychiatric diagnostics. Neurosci Biobehav Rev. (2015) 57:328–49. 10.1016/j.neubiorev.2015.08.001 26254595

[11] Pomponio R Erus G Habes M Doshi J Srinivasan D Mamourian E. Harmonization of large MRI datasets for the analysis of brain imaging patterns throughout the lifespan. Neuroimage. (2020) 208:116450. 10.1016/j.neuroimage.2019.116450 31821869 PMC6980790

[12] Schmidt MF Storrs JM Freeman KB Jack CR Turner ST Griswold ME. A comparison of manual tracing and FreeSurfer for estimating hippocampal volume over the adult lifespan. Hum Brain Mapp. (2018) 39:2500. 10.1002/hbm.24017 29468773 PMC5951757

[13] Tullo S Patel R Devenyi GA Salaciak A Bedford SA Farzin S. MR-based age-related effects on the striatum, globus pallidus, and thalamus in healthy individuals across the adult lifespan. Hum Brain Mapp. (2019) 40:5269. 10.1002/hbm.24771 31452289 PMC6864890

[14] Solanes A Palau P Fortea L Salvador R González-Navarro L Llach CD. Biased accuracy in multisite machine-learning studies due to incomplete removal of the effects of the site. Psychiatry Res Neuroimaging. (2021) 314:e111313. 10.1016/j.pscychresns.2021.111313 34098248

[15] Gronenschild EHBM Gronenschild EHB Habets P Jacobs HIL Mengelers R. The effects of freesurfer version, workstation type, and macintosh operating system version on anatomical volume and cortical thickness measurements. PLoS ONE. (2012) 7:e38234. 10.1371/journal.pone.0038234 22675527 PMC3365894

[16] Jovicich J Czanner S Han X Salat D van der Kouwe A Quinn B. MRI-derived measurements of human subcortical, ventricular and intracranial brain volumes: reliability effects of scan sessions, acquisition sequences, data analyses, scanner upgrade, scanner vendors and field strengths. Neuroimage. (2009) 46:177–192. 10.1016/j.neuroimage.2009.02.010 19233293 PMC2866077

[17] Panman JL To YY van der Ende EL Poos JM Jiskoot LC Meeter LHH. Bias introduced by multiple head coils in MRI research: an 8 channel and 32 channel coil comparison. Front Neurosci. (2019) 13:729. 10.3389/fnins.2019.00729 31379483 PMC6648353

[18] Zaitsev M Maclaren J Herbst M. Motion artefacts in MRI: a complex problem with many partial solutions. J Magn Reson Imaging. (2015) 42:887. 10.1002/jmri.24850 25630632 PMC4517972

[19] Jezzard P. Correction of geometric distortion in fMRI data. Neuroimage. (2012) 62:648–51. 10.1016/j.neuroimage.2011.09.010 21945795

[20] Iskurt A Becerikli Y Mahmutyazicioglu K. Automatic identification of landmarks for standard slice positioning in brain MRI. J Magnetic Resonance Imaging. (2011) 34:499–510. 10.1002/jmri.22717 21751290

[21] van der Kouwe AJW Benner T Fischl B Schmitt F Salat DH Harder M. On-line automatic slice positioning for brain MR imaging. Neuroimage. (2005) 27:222–30. 10.1016/j.neuroimage.2005.03.035 15886023

[22] Kawagoe T Onoda K Yamaguchi S. Different pre-scanning instructions induce distinct psychological and resting brain states during functional magnetic resonance imaging. Eur J Neurosci. (2018) 47:77–82. 10.1111/ejn.13787 29205574

[23] Sämann PG Iglesias JE Gutman B Grotegerd D Leenings R Flint C. FreeSurfer-based segmentation of hippocampal subfields: a review of methods and applications, with a novel quality control procedure for ENIGMA studies and other collaborative efforts. Hum Brain Mapp. (2020). 10.31234/osf.io/uhwtk 33368865 PMC8805696

[24] Waller L Erk S Pozzi E Toenders YJ Haswell CC Büttner M. ENIGMA HALFpipe: Interactive, reproducible, and efficient analysis for resting-state and task-based fMRI data. Hum Brain Mapp. (2022) 43:2727–42. 10.1002/hbm.25829 35305030 PMC9120555

[25] Ashburner J. A fast diffeomorphic image registration algorithm. Neuroimage. (2007) 38:95–113. 10.1016/j.neuroimage.2007.07.007 17761438

[26] Ashburner J Friston KJ. Why voxel-based morphometry should be used. Neuroimage. (2001) 14:1238–43. 10.1006/nimg.2001.0961 11707080

[27] Ashburner J Friston KJ. Unified segmentation. Neuroimage. (2005) 26:839–51. 10.1016/j.neuroimage.2005.02.018 15955494

[28] Littlejohns TJ Holliday J Gibson LM Garratt S Oesingmann N Alfaro-Almagro F. The UK Biobank imaging enhancement of 100,000 participants: rationale, data collection, management and future directions. Nat Commun. (2020) 11:2624. 10.1038/s41467-020-15948-9 32457287 PMC7250878

[29] Brückl TM Spoormaker VI Sämann PG Brem AK Henco L. The biological classification of mental disorders (BeCOME) study: a protocol for an observational deep-phenotyping study for the identification of biological subtypes. BMC Psychiatry. (2020) 20:213. 10.1186/s12888-020-02541-z 32393358 PMC7216390

[30] Smitha KA Akhil RK Arun KM Rajesh PG Thomas B Kapilamoorthy TR. Resting state fMRI: a review on methods in resting state connectivity analysis and resting state networks. Neuroradiol J. (2017) 30:e7342. 10.1177/1971400917697342 28353416 PMC5524274

[31] Bullmore E Sporns O. Complex brain networks: graph theoretical analysis of structural and functional systems. Nat Rev Neurosci. (2009) 10:e2575. 10.1038/nrn2575 19190637

[32] Fortin JP Parker D Tunç B Watanabe T Elliott MA. Harmonization of multi-site diffusion tensor imaging data. Neuroimage. (2017) 161:149–70. 10.1016/j.neuroimage.2017.08.047 28826946 PMC5736019

[33] Fortin JP Cullen N Sheline YI Taylor WD Aselcioglu I Cook PA. Harmonization of cortical thickness measurements across scanners and sites. Neuroimage. (2018) 167:e024. 10.1016/j.neuroimage.2017.11.024 29155184 PMC5845848

[34] Johnson WE Li C Rabinovic A. Adjusting batch effects in microarray expression data using empirical Bayes methods. Biostatistics. (2007) 8:118–27. 10.1093/biostatistics/kxj037 16632515

[35] Chen AA Beer JC Tustison NJ Cook PA Shinohara RT Shou H. Mitigating site effects in covariance for machine learning in neuroimaging data. Hum Brain Mapp. (2021) 43:1179–95. 10.1002/hbm.25688 34904312 PMC8837590

[36] Beer JC Tustison NJ Cook PA Davatzikos C Sheline YI Shinohara RT. Longitudinal ComBat: a method for harmonizing longitudinal multi-scanner imaging data. Neuroimage. (2020) 220:117129. 10.1016/j.neuroimage.2020.117129 32640273 PMC7605103

[37] Garcia-Dias R Scarpazza C Baecker L Vieira S Pinaya WHL Corvin A. Neuroharmony: a new tool for harmonizing volumetric MRI data from unseen scanners. Neuroimage. (2020) 220:117127. 10.1016/j.neuroimage.2020.117127 32634595 PMC7573655

[38] Bayer JMM Dinga R Kia SM Kottaram AR Wolfers T Lv J. Accommodating site variation in neuroimaging data using normative and hierarchical Bayesian models. bioRxiv. (2021). 10.1101/2021.02.09.430363 PMC7614761 36272672

[39] Kia SM Huijsdens H Dinga R Wolfers T Mennes M Andreassen OA. Hierarchical bayesian regression for multi-site normative modeling of neuroimaging data. Med Image Comput Computer Assisted Intervention. (2020) 2020:699–709. 10.1007/978-3-030-59728-3_68

[40] Kia SM Huijsdens H Rutherford S Dinga R Wolfers T Mennes M. Federated Multi-Site Normative Modeling using Hierarchical Bayesian Regression. *BioRxiv [Preprint].* (2021). 10.1101/2021.05.28.446120 PMC9731431 36480551

[41] Moyer D Ver Steeg G Tax CMW Thompson PM. Scanner invariant representations for diffusion MRI harmonization. Magn Reson Med. (2020) 84:e28243. 10.1002/mrm.28243 32250475 PMC7384065

[42] Sinha S Thomopoulos SI Lam P Muir A Thompson PM. Alzheimer's Disease Classification Accuracy is Improved by MRI Harmonization Based on Attention-Guided Generative Adversarial Networks. BioRxiv [Preprint]. (2021). 10.1101/2021.07.26.453862 PMC8952312 35340753

[43] Dewey BE Zuo L Carass A He Y Liu Y Mowry EM. A disentangled latent space for cross-site MRI harmonization. Medical Image Comput Comp Assisted Intervention. (2020) 2020:720–9. 10.1007/978-3-030-59728-3_70

[44] Liu M Maiti P Thomopoulos S Zhu A Chai Y Kim H. Style transfer using generative adversarial networks for multi-site mri harmonization. In: International Conference on Medical Image Computing and Computer-Assisted Intervention. Cham: Springer (2021). p. 313–21. 10.1007/978-3-030-87199-4_30 PMC9137427 35647615

[45] Leek JT Storey JD. Capturing heterogeneity in gene expression studies by surrogate variable analysis. PLoS Genet. (2007) 3:1724–35. 10.1371/journal.pgen.0030161 17907809 PMC1994707

[46] Nygaard V Rødland EA Hovig E. Methods that remove batch effects while retaining group differences may lead to exaggerated confidence in downstream analyses. Biostatistics. (2016) 17:29–39. 10.1093/biostatistics/kxv027 26272994 PMC4679072

[47] Parmigiani G Garrett ES Irizarry RA Zeger SL. The analysis of gene expression data: an overview of methods and software. Statist Biol Health. (2003) 2003:1–45. 10.1007/0-387-21679-0_1

[48] Orlhac F Eertink JJ Cottereau AS Zijlstra JM Thieblemont C. A guide to ComBat harmonization of imaging biomarkers in multicenter studies. J Nucl Med. (2021) 63:172–9. 10.2967/jnumed.121.262464 34531263 PMC8805779

[49] Chen AA Beer JC Tustison NJ Cook PA Shinohara RT Shou H. Removal of scanner effects in covariance improves multivariate pattern analysis in neuroimaging data. BioRxiv [Preprint]. (2020).

[50] Dempster AP Laird NM Rubin DB. Maximum likelihood from incomplete data via the em algorithm. J Royal Stat Soci. Series B (Methodol). (1977) 39:1–38. Available online at: http://www.jstor.org/stable/2984875

[51] Radua J Vieta E Shinohara R Kochunov P Quidé Y Green MJ. Increased power by harmonizing structural MRI site differences with the ComBat batch adjustment method in ENIGMA. Neuroimage. (2020) 218:e116956. 10.1016/j.neuroimage.2020.116956 32470572 PMC7524039

[52] Stein CK Qu P Epstein J Buros A Rosenthal A Crowley J. Removing batch effects from purified plasma cell gene expression microarrays with modified ComBat. BMC Bioinform. (2015) 16:63. 10.1186/s12859-015-0478-3 25887219 PMC4355992

[53] Marquand AF Kia SM Zabihi M Wolfers T Buitelaar JK Beckmann CF. Conceptualizing mental disorders as deviations from normative functioning. Mol Psychiatry. (2019) 24:1415–24. 10.1038/s41380-019-0441-1 31201374 PMC6756106

[54] Wood SN. Generalized Additive Models: An Introduction With R, Second Edition. CRC Press (2017). 10.1201/9781315370279

[55] Da-Ano R Masson I Lucia F Doré M Robin P Alfieri J. Performance comparison of modified ComBat for harmonization of radiomic features for multicenter studies. Sci Rep. (2020) 10:10248. 10.1038/s41598-020-66110-w 32581221 PMC7314795

[56] Bethlehem RAI Seidlitz J Romero-Garcia R. Using normative age modelling to isolate subsets of individuals with autism expressing highly age-atypical cortical thickness features. bioRxiv. [Preprint]. (2018) 1–21. 10.1101/252593

[57] Wolfers T Doan NT Kaufmann T Alnæs D Moberget T Agartz I. Mapping the heterogeneous phenotype of schizophrenia and bipolar disorder using normative models. JAMA Psychiatry. (2018) 75:1146–55. 10.1001/jamapsychiatry.2018.2467 30304337 PMC6248110

[58] Wolfers T Beckmann CF Hoogman M Buitelaar JK Franke B Marquand AF. Individual differences vs. the average patient: mapping the heterogeneity in ADHD using normative models. Psychol Med. (2020) 50:314–23. 10.1017/S0033291719000084 30782224 PMC7083555

[59] Borghi E de Onis M Garza C Van den Broeck J Frongillo EA Grummer-Strawn L. Construction of the World Health Organization child growth standards: selection of methods for attained growth curves. Stat Med. (2006) 25:247–65. 10.1002/sim.2227 16143968

[60] Fraza CJ Dinga R Beckmann CF Marquand AF. Warped Bayesian linear regression for normative modelling of big data. Neuroimage. (2021) 245:118715. 10.1016/j.neuroimage.2021.118715 34798518 PMC7613680

[61] Lv J Di Biase M Cash RFH Cocchi L Cropley VL Klauser P. Individual deviations from normative models of brain structure in a large cross-sectional schizophrenia cohort. Mol Psychiatry. (2021) 26:3512–23. 10.1038/s41380-020-00882-5 32963336 PMC8329928

[62] Dinga R Fraza CJ Bayer JMM Kia SM Beckmann CF Marquand AF. Normative Modeling of neuroimaging Data Using Generalized Additive Models Of Location Scale and Shape. BioRxiv [Preprint]. (2021). 10.1101/2021.06.14.448106

[63] Dewey BE Zhao C Reinhold JC Carass A Fitzgerald KC Sotirchos ES. DeepHarmony: a deep learning approach to contrast harmonization across scanner changes. Magn Reson Imaging. (2019) 64:160–70. 10.1016/j.mri.2019.05.041 31301354 PMC6874910

[64] Zhao F Wu Z Wang L Lin W Xia S Shen D. Harmonization of infant cortical thickness using surface-to-surface cycle-consistent adversarial networks. Med Image Comput Assist Interv. (2019) 11767:475–83. 10.1007/978-3-030-32251-9_52 32128523 PMC7052700

[65] Goodfellow I Bengio Y Courville A. Deep Learning. MIT Press (2016).

[66] Zhao Q Adeli E Pohl KM. Training confounder-free deep learning models for medical applications. Nat Commun. (2020) 11:6010. 10.1038/s41467-020-19784-9 33243992 PMC7691500

[67] Dinsdale NK Jenkinson M Namburete AIL. Deep learning-based unlearning of dataset bias for MRI harmonisation and confound removal. Neuroimage. (2021) 228:117689. 10.1016/j.neuroimage.2020.117689 33385551 PMC7903160

[68] Huang X Liu MY Belongie S Kautz J. Multimodal unsupervised image-to-image translation. Computer Vision. (2018) 2018:179–96. 10.1007/978-3-030-01219-9_11

[69] Zuo L Dewey BE Liu Y He Y Newsome SD Mowry EM. Unsupervised MR harmonization by learning disentangled representations using information bottleneck theory. Neuroimage. (2021) 243:118569. 10.1016/j.neuroimage.2021.118569 34506916 PMC10473284

[70] Lee HY Tseng HY Mao Q Huang B Lu YD Singh M. DRIT: diverse image-to-image translation *via* disentangled representations. Int J Comput Vis. (2020) 128:2402–17. 10.1007/s11263-019-01284-z

[71] Liu S Yap P-T. Learning multi-site harmonization of magnetic resonance images without traveling human phantoms. Arxiv. [Preprint]. (2021) 1–16. 10.48550/arXiv.2110.00041 PMC10898625 38420332

[72] Rao A Monteiro JM Mourao-Miranda J. Predictive modelling using neuroimaging data in the presence of confounds. Neuroimage. (2017) 150:23–49. 10.1016/j.neuroimage.2017.01.066 28143776 PMC5391990

[73] Zhang Y Jenkins DF Manimaran S Evan Johnson W. Alternative empirical Bayes models for adjusting for batch effects in genomic studies. BMC Bioinformatics. (2018) 19:e2263. 10.1186/s12859-018-2263-6 30001694 PMC6044013

[74] de Wit SJ Alonso P Schweren L Mataix-Cols D Lochner C Menchón JM. Multicenter voxel-based morphometry mega-analysis of structural brain scans in obsessive-compulsive disorder. Am J Psychiatry. (2014) 171:340–9. 10.1176/appi.ajp.2013.13040574 24220667

[75] Sun D Rakesh G Haswell CC Logue M Baird CL O'Leary EM. A comparison of methods to harmonize cortical thickness measurements across scanners and sites. Neuroimage. (2022) 261:119509. 10.1016/j.neuroimage.2022.119509 35917919 PMC9648725

[76] Orlhac F Lecler A Savatovski J Goya-Outi J Nioche C Charbonneau F. How can we combat multicenter variability in MR radiomics? Validation of a correction procedure. Eur Radiol. (2021) 31:2272–80. 10.1007/s00330-020-07284-9 32975661

[77] Mietchen D Gaser C. Computational morphometry for detecting changes in brain structure due to development, aging, learning, disease and evolution. Front Neuroinform. (2009) 3:e2009. 10.3389/neuro.11.025.2009 19707517 PMC2729663

[78] Reuter M Schmansky NJ Rosas HD Fischl B. Within-subject template estimation for unbiased longitudinal image analysis. Neuroimage. (2012) 61:1402–18. 10.1016/j.neuroimage.2012.02.084 22430496 PMC3389460

[79] DuPre E Spreng RN. Structural covariance networks across the life span, from 6 to 94 years of age. Netw Neurosci. (2017) 1:302–23. 10.1162/NETN_a_00016 29855624 PMC5874135

[80] Seeley WW Crawford RK Zhou J Miller BL Greicius MD. Neurodegenerative diseases target large-scale human brain networks. Neuron. (2009) 62:42–52. 10.1016/j.neuron.2009.03.024 19376066 PMC2691647

[81] Da-Ano R Lucia F Masson I Abgral R Alfieri J Rousseau C. A transfer learning approach to facilitate ComBat-based harmonization of multicentre radiomic features in new datasets. PLoS ONE. (2021) 16:e0253653. 10.1371/journal.pone.0253653 34197503 PMC8248970

[82] Gössl C Auer DP Fahrmeir L. Bayesian spatiotemporal inference in functional magnetic resonance imaging. Biometrics. (2001) 57:554–62. 10.1111/j.0006-341X.2001.00554.x 11414583

[83] Tax CM Grussu F Kaden E Ning L Rudrapatna U Evans CJ. Cross-scanner and cross-protocol diffusion MRI data harmonisation: a benchmark database and evaluation of algorithms. NeuroImage. (2019) 195:285–99. 10.1016/j.neuroimage.2019.01.077 30716459 PMC6556555

