# Supplementary material for: Site effects how-to and when: An overview of retrospective techniques to accommodate site effects in multi-site neuroimaging analyses
Source: Front Neurol. 2022 Oct 31;13:923988. doi: 10.3389/fneur.2022.923988 (PMC9661923; doi:10.3389/fneur.2022.923988)
Supplement: Supplementary file 1 [file Data_Sheet_1.PDF]

**APPENDIX**

*Detailed derivation of the estimates for the posterior conditionals (parametric)*

In the following, we describe the estimate for posterior conditional $\gamma_{sf}^{*} = \pi(\gamma_{sf}|Y_{sf}, \delta_{sf}^{2})$ from prior and posterior parametric distributions of $\gamma_{sf}$ followed by the derivation of the posterior conditional from $\delta_{sf}^{2*} = \pi(\delta_{sf}^{2}|Y_{sf}\gamma_{sf})$ from prior and posterior parametric distributions of $\delta_{sf}^{2}$.

We assume the prior distribution of $\gamma_{sf}$ to follow $\gamma_{sf} \sim N(\hat{\gamma}_s, \tau_s^2)$ and we apply Bayes' Theorem to find the posterior conditional distribution of $\gamma_{sf}$ denoted $\pi(\gamma_{sf}|Y_{sf}, \delta_{sf}^{2})$ which is proportional to the fusion between the likelihood and the prior:

$$\pi(\gamma_{sf}|Y_{sf}, \delta_{sf}^{2}) \propto L(Y_{sf}|\gamma_{sf}, \delta_{sf}^{2})\pi(\gamma_{sf})$$

$$\propto exp\{-\frac{1}{2\delta_{sf}^{2}}\sum_{i}(Y_{isf}-\gamma_{sf})^2\ \}exp\{-\frac{1}{2\tau_s^2}(\gamma_{sf}-\gamma_s)^2\}$$

$$= exp\{-\frac{1}{2\delta_{sf}^{2}}[\sum_{i}Y_{isf}^2 - 2\sum_{i}Y_{isf}\gamma_{sf} + n_s\gamma_{sf})^2] - \frac{1}{2\tau_s^2}[\gamma_{sf} - 2\gamma_{sf}\gamma_s + \gamma_s^2]\}$$

$$\propto exp\{-\frac{1}{2}\ (\frac{n_s\tau_s^2 + \delta_{sf}^2}{\delta_{sf}^{2\tau_s^2}})\ [\gamma_{sf}^2 - 2(\frac{\tau_s^2\sum_i Y_{isf} + \delta_{sf}^2\gamma_s}{n_s\tau_s^2 + \delta_{sf}^2)})\gamma_{sf}]\}$$

By completing the square, we get:

$$exp\{-\frac{1}{2}\ (\frac{n_s\tau_s^2 + \delta_{sf}^2}{\delta_{sf}^2\tau_s^2})\ [\gamma_{sf} - (\frac{\tau_s^2\sum_i Y_{isf} + \delta_{sf}^2\gamma_s}{n_s\tau_s^2 + \delta_{sf}^2)})]^2\}$$

, which describes the kernel of a normal distribution with expected value:

$$E[\gamma_{sf}|Y_{sf}, \sigma_{sf}^2] = \frac{\tau_s^2\sum_i Y_{isf} + \delta_{sf}^2\gamma_s}{n_s\tau_s^2 + \delta_{sf}^2)}$$

which, given $\hat{\gamma}_{sf}, \hat{\delta}_{sf}^2, \bar{\gamma}_s$ and $\bar{\tau_s}^2$ as set above, can be re-written to give the following estimate $\gamma_{sf}^{*}$:

$$\gamma_{sf}^{*} = \hat{E}[\gamma_{sf}|Y_{sf}, \sigma_{sf}^{2*}] = \frac{n_s\bar{\tau}_s^2\hat{\gamma}_{sf} + \delta_{sf}^2\bar{\gamma}_s}{n_s\bar{\tau}_s^2 + \delta_{sf}^{2*}}$$

.

Next, we describe the derivation of the posterior conditional from $\delta_{sf}^{2*} = \pi(\delta_{sf}^{2}|Y_{sf}\gamma_{sf})$ from prior and posterior parametric distributions of $\delta_{sf}^{2}$.

For the posterior conditional distribution of $\delta^2_{sf}$, given the *Inverse* $\Gamma(\lambda_s, \theta_s)$ prior, we set:

$$\pi(\delta^2_{sf}|Y_{sf}\gamma_{sf}) \propto L(Y_{sf}|\gamma_{sf}\delta^2_{sf})\pi(\delta^2_{sf})$$

$$\propto (\delta_{sf})^{\frac{-n_i}{2}} exp\{-\frac{1}{2\delta^2_{sf}}\sum_i (Y_{isf} - \gamma_{sf})^2\}(\delta^2_{sf})^{-\lambda_i+1}\ exp\{-\frac{\theta_i}{\delta^2_{sf}}\}$$

$$= (\delta_{sf})^{-(\frac{n_i}{2}+\lambda_s)-1} exp\{-\frac{\theta_s + \frac{1}{2}\sum_i (Y_{isf} - \gamma_{sf})^2}{\delta^2_{sf}}\}.$$

This describes an *Inverse* $\Gamma$ distribution with the expected value:

$$E[\delta^2_{sf}|Y_{sf}, \gamma_{sf}] = \frac{\theta_s + \frac{1}{2}\sum_i (Y_{isf} - \gamma_{sf})^2}{\frac{n_i}{2} + \lambda_s - 1}$$

Subsetting this equation with the Method of Moments estimates for $\bar{\theta}_s$ and $\bar{\lambda}_s$, we get the estimate for $\delta^{2*}_{sf}$ :

$$\delta^{2*}_{sf} = \hat{E}[\delta^2_{sf}|Y_{sf}, \gamma^*_{sf}] = \frac{\bar{\theta}_s + \frac{1}{2}\sum_i (Y_{isf} - \gamma^*_{sf})^2}{\frac{n_i}{2} + \bar{\lambda}_s - 1}$$
