# Supplementary material for: Site effects how-to and when: An overview of retrospective techniques to accommodate site effects in multi-site neuroimaging analyses
Source: Front Neurol. 2022 Oct 31;13:923988. doi: 10.3389/fneur.2022.923988 (PMC9661923; doi:10.3389/fneur.2022.923988)
Supplement: Supplementary file 2 [file Table_1.docx]

| Method/  Algorithms | Paper cited in manuscript | Feature/Imaging modality | Number of individuals, sample type | Number of sites/batches |
| --- | --- | --- | --- | --- |
| ComBat | Johnson & Li (2000) [1] | Oligonucleotide microarray data | Data set 1: 628 genes, 12 samples (4 experiment, 8 control)  Data set 2: NA genes, 30 samples, (18 experiment, 12 controls) | 3 batches  3 batches |
|  | Fortin et al. (2017) [2] | Diffusion tensor imaging (Fractional Anisotropy, Mean Diffusivity, voxel-wise) | - **Discovery cohort**: 210 healthy participants from 8 to 19 years - **Validation cohort** 397 healthy participants from 8 to 19 years | 2 sites (Discovery cohort)  2 sites (Validation cohort) |
|  | Fortin et al. (2018) [3] | Thickness measures of 98 cortical regions | Independent application to 2 different data sets:   - 200 individuals with depression, 40 healthy individuals from 18–65 years - 177 individuals with depression and 59 healthy controls, from 58–95 years | Data set 1: 4 sites  Data set 2: 7 sites |
|  | Radua et al. (2020) [4] | 70 cortical and subcortical ROIS, 10 subcortical volumes ROIS | 3041 healthy controls (mean 33.9 [SD 12.0] years), 2897 individuals with schizophrenia (mean 33.3 [13.2] years). | 33 sites |
| Distributed ComBat | Chen et al. (2022) [5] | 62 Cortical thickness ROIS | 505 subjects, 115 Alzheimer’s disease patients, 239 late mild cognitive impairment, and 151 cognitively normal | 64 scanners, 53 sites |
| Modified ComBat | Da-ano et al. (2020) [6] | Radiomics: PET/CT/MRI (T2- weighted, diffusion-weighted) | 197 individuals with cervical cancer  98 patients with laryngeal cancer | 3 sites  5 sites |
| Bootstrap ComBat | Da-ano et al. (2020) [6] | Radiomics: PET/CT/MRI (T2- weighted, diffusion-weighted) | 197 individuals with cervical cancer  98 patients with laryngeal cancer | 3 sites  5 sites |
| ComBat-GAM | Pomponio et al. (2019) [7], [8] | Cortical thickness, surface area and subcortical volumes data (145 ROIs) | 10,232 in total, age range 8-89 years | 18 sites |
| Longitudinal ComBat | Beer et al. (2020) [9] | Cortical thickness data of 62 ROIS | 197 cognitively normal, 324 late mild cognitive impairment, 142 Alzheimer’s disease, 663 individuals in total, age range from 55-92 years | 58 sites, 126 scanners |
| CovBat | Chen et al. (2021) [10] | 62 Cortical thickness ROIS | 505 subjects, 115 Alzheimer’s disease patients, 239 late mild cognitive impairment, and 151 cognitively normal | 64 scanners, 53 sites |
| Normative modeling | Bayer et al. (2021) [11] | Cortical thickness (35 bilateral ROIs) | 569 healthy controls and 482 individuals with autism spectrum disorder, 1051 total, age range 7-40 years | 20 sites |
|  | Kia et al. (2020) [12] | cortical thickness (148 cortical ROIS) | 7499 scans, 1017 patients (attention deficit hyperactivity disorder, Alzheimer’s disease, mild cognitive impairment, schizophrenia, bipolar disorder, depression), from 8 to 97 years | 7 data sets, 33 sites |
|  | Kia et al. (2021) [13] | cortical thickness (148 cortical ROIS) | 37126 scans, 1107 patients, (attention deficit hyperactivity disorder, Alzheimer’s disease, mild cognitive impairment, schizophrenia, bipolar disorder, depression, early psychosis), 6-100 years | 79 sites |
| Neuroharmony | Rafael Garcia-Dias et al. (2019) [14] | Volumes of 101 ROIs | 15,026 subjects including healthy controls and individuals with various mental disorders, age range 10-80 years | 62 sites (32 datasets) |
| Deep learning | **VAE-GAN**  Moyer et al.,  (2020) [15] | Raw T1 weighted images | 10 subjects, no demographics provided | 4 sites (2 scanners * 2 protocols) |
|  | **CycleGAN**  Zhao et al. (2019) [16] | Cortical thickness and surface maps | 360 scans from 183 infants, age from 0 to 2 years | Two sites, one created from re-sampling the data |
|  | Dewey et al. (2020) [17] | T1 and T2 weighted raw images | 30 training subjects, 10 validation subjects and 10 testing subjects per site | 3 sites |
|  | **CALAMITI**  Zuo et al. (2021)[18] | T1 and T2 weighted raw images | Total number of subjects not provided | 3 data sets, 10 sites |
|  | **MURD**  Liu & Yap (2021) [19] | T1 and T2 weighted raw images | 20 training subjects, 10 validation subjects, 1000 generalisation subjects, 1 travelling subject per site, age range 9-10 years | 3 sites |
| S1. Features, validation population characteristics and number of batches per method. | | | | |

[1] W. E. Johnson, C. Li, and A. Rabinovic, “Adjusting batch effects in microarray expression data using empirical Bayes methods,” *Biostatistics*, vol. 8, no. 1, pp. 118–127, 2007, doi: 10.1093/biostatistics/kxj037.

[2] J. P. Fortin *et al.*, “Harmonization of multi-site diffusion tensor imaging data,” *Neuroimage*, vol. 161, pp. 149–170, 2017, doi: 10.1016/j.neuroimage.2017.08.047.

[3] J. P. Fortin *et al.*, “Harmonization of cortical thickness measurements across scanners and sites,” *Neuroimage*, vol. 167, pp. 104–120, 2018, doi: 10.1016/j.neuroimage.2017.11.024.

[4] J. Radua *et al.*, “Increased power by harmonizing structural MRI site differences with the ComBat batch adjustment method in ENIGMA,” *Neuroimage*, vol. 218, p. 116956, 2020, doi: 10.1016/j.neuroimage.2020.116956.

[5] A. A. Chen, C. Luo, Y. Chen, R. T. Shinohara, and H. Shou, “Privacy-preserving harmonization via distributed ComBat,” *Biorxiv*, p. 2021.07.30.454516, 2021, doi: 10.1101/2021.07.30.454516.

[6] R. Da-ano *et al.*, “Performance comparison of modified ComBat for harmonization of radiomic features for multicenter studies,” *Sci Rep-uk*, vol. 10, no. 1, p. 10248, 2020, doi: 10.1038/s41598-020-66110-w.

[7] R. Pomponio *et al.*, “Harmonization of large multi-site imaging datasets: Application to 10,232 MRIs for the analysis of imaging patterns of structural brain change throughout the lifespan,” *Biorxiv*, p. 784363, 2019, doi: 10.1101/784363.

[8] R. Pomponio *et al.*, “Harmonization of large MRI datasets for the analysis of brain imaging patterns throughout the lifespan,” *Neuroimage*, vol. 208, p. 116450, 2020, doi: 10.1016/j.neuroimage.2019.116450.

[9] J. C. Beer *et al.*, “Longitudinal ComBat: A method for harmonizing longitudinal multi-scanner imaging data,” *Neuroimage*, vol. 220, p. 117129, 2020, doi: 10.1016/j.neuroimage.2020.117129.

[10] A. A. Chen *et al.*, “Mitigating site effects in covariance for machine learning in neuroimaging data,” *Hum Brain Mapp*, 2021, doi: 10.1002/hbm.25688.

[11] J. M. M. Bayer *et al.*, “Accommodating Site Variation in Neuroimaging Data Using Hierarchical and Bayesian Models,” *bioRxiv*, 2021.

[12] S. M. Kia *et al.*, “Hierarchical Bayesian Regression for Multi-Site Normative Modeling of Neuroimaging Data,” *Arxiv*, 2020.

[13] S. M. Kia *et al.*, “Federated Multi-Site Normative Modeling using Hierarchical Bayesian Regression,” *Biorxiv*, p. 2021.05.28.446120, 2021, doi: 10.1101/2021.05.28.446120.

[14] R. Garcia-Dias *et al.*, “Neuroharmony: A new tool for harmonizing volumetric MRI data from unseen scanners,” *Neuroimage*, vol. 220, p. 117127, Oct. 2020, doi: 10.1016/j.neuroimage.2020.117127.

[15] D. Moyer, G. V. Steeg, C. M. W. Tax, and P. M. Thompson, “Scanner Invariant Representations for Diffusion MRI Harmonization,” *Arxiv*, 2019.

[16] K. Zhao *et al.*, “Cortical thickness and subcortical structure volume abnormalities in patients with major depression with and without anxious symptoms,” *Brain Behav*, vol. 7, no. 8, p. e00754, 2017, doi: 10.1002/brb3.754.

[17] B. E. Dewey *et al.*, “DeepHarmony: A deep learning approach to contrast harmonization across scanner changes,” *Magn Reson Imaging*, vol. 64, pp. 160–170, 2019, doi: 10.1016/j.mri.2019.05.041.

[18] L. Zuo *et al.*, “Unsupervised MR harmonization by learning disentangled representations using information bottleneck theory,” *Neuroimage*, vol. 243, p. 118569, 2021, doi: 10.1016/j.neuroimage.2021.118569.

[19] S. Liu and P.-T. Yap, “Learning Multi-Site Harmonization of Magnetic Resonance Images Without Traveling Human Phantoms,” *Arxiv*, 2021.
